# Microcarriers for Upscaling Cultured Meat Production

**DOI:** 10.3389/fnut.2020.00010

**Published:** 2020-02-20

**Authors:** Vincent Bodiou, Panagiota Moutsatsou, Mark J. Post

**Affiliations:** ^1^Department of Physiology, Faculty of Health, Medicine and Life Sciences, School for Cardiovascular Diseases, Maastricht University, Maastricht, Netherlands; ^2^Mosa Meat BV, Maastricht, Netherlands; ^3^CARIM, Faculty of Health, Medicine and Life Sciences, School for Cardiovascular Diseases, Maastricht University, Maastricht, Netherlands

**Keywords:** cultivated meat, clean meat, bovine myoblasts, satellite cells, bioprocessing, microbeads, cell expansion

## Abstract

Due to the considerable environmental impact and the controversial animal welfare associated with industrial meat production, combined with the ever-increasing global population and demand for meat products, sustainable production alternatives are indispensable. In 2013, the world's first laboratory grown hamburger made from cultured muscle cells was developed. However, coming at a price of $300.000, and being produced manually, substantial effort is still required to reach sustainable large-scale production. One of the main challenges is scalability. Microcarriers (MCs), offering a large surface/volume ratio, are the most promising candidates for upscaling muscle cell culture. However, although many MCs have been developed for cell lines and stem cells typically used in the medical field, none have been specifically developed for muscle stem cells and meat production. This paper aims to discuss the MCs' design criteria for skeletal muscle cell proliferation and subsequently for meat production based on three scenarios: (1) MCs are serving only as a temporary substrate for cell attachment and proliferation and therefore they need to be separated from the cells at some stage of the bioprocess, (2) MCs serve as a temporary substrate for cell proliferation but are degraded or dissolved during the bioprocess, and (3) MCs are embedded in the final product and therefore need to be edible. The particularities of each of these three bioprocesses will be discussed from the perspective of MCs as well as the feasibility of a one-step bioprocess. Each scenario presents advantages and drawbacks, which are discussed in detail, nevertheless the third scenario appears to be the most promising one for a production process. Indeed, using an edible material can limit or completely eliminate dissociation/degradation/separation steps and even promote organoleptic qualities when embedded in the final product. Edible microcarriers could also be used as a temporary substrate similarly to scenarios 1 and 2, which would limit the risk of non-edible residues.

## Introduction

The livestock sector is responsible for 18% of greenhouse gas emissions, 8% of human water consumption and contributes to water, air and soil pollution (1). Taking into account the predicted global population increase for 2050 (2) and the ever increasing meat consumption (3), sustainable alternatives are urgently needed. Since the first laboratory grown hamburger in 2013, research on cultured meat has taken off all around the world. Its potential to reduce the environmental impact and eliminate the controversial treatment of animals (4) associated with industrial meat production has attracted a vast interest. Different life cycle analyses for cultured meat production have been theorized. Mattick's et al. (5) study presents significant differences between different types of meat. For instance, pork and poultry produced by cellular agriculture technology would lead to higher global warming potential, whereas beef would lead to a lower impact (5). Other long-term, worst case scenario models predict an initially greater peak warming due to cattle as opposed to cultured meat, but a higher warming effect of cultured meat in the long run, due to the different way that CO_2_ and CH_4_ gases accumulate in the atmosphere. However, these studies use current methods of energy production in their models not taking into account potential energy decarbonization for the next 1,000 years (6). Smetana's et al. (7) study also shows a direct link between environmental impact and method of energy production, as cultured meat processing is highly energy dependent (7). It is therefore possible that innovation in the energy field will result in decarbonization of energy and thus lead to a more sustainable process than the one expected by the less optimistic models. Cultured meat also has the possibility to improve consumer health and nutrition by tailoring product composition (8) and to reduce zoonotic contamination by working under controlled atmosphere, compared to poor handling and hygiene in animal farming (9, 10). However, mainly due to the astronomical production costs, substantial effort is still required to reach sustainable and cost-effective large-scale production.

Several methods of producing cultured meat have been proposed and different cell types have been considered, including embryonic stem cells (ESCs), induced pluripotent stem cells (IPSCs), mesenchymal stem cells (MSCs) and satellite cells (SCs) (8, 11–13), The latter, also called bovine muscle stem cells, seem the most straight-forward, suitable candidates for this purpose. They are mononuclear cells which can be found between the basal membrane and the sarcolemma of nearby muscle fibers in mammalian's skeletal muscles (14). They are involved in skeletal muscle regeneration and have the ability to proliferate while keeping their stemness and, when specific signaling pathways are activated, they can differentiate into muscle cells. As opposed to IPSCs, MSCs, and ESCs which can differentiate into different lineages, SCs can only differentiate into myocytes, thus facilitating the whole bioprocess.

**Figure 1:**
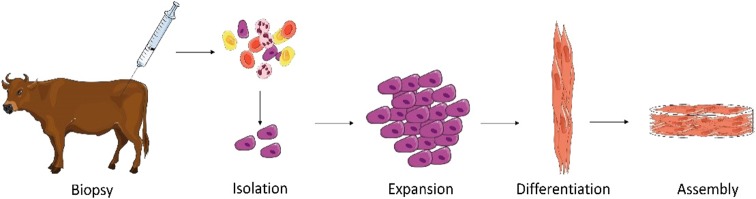
Main steps required for production of cultured meat from an animal biopsy. A muscle biopsy is performed on a living animal. Satellite cells (SCs) are then isolated and subsequently expanded. When a sufficient quantity of cells is obtained, differentiation of SCs is induced. This includes cell fusion and myotube formation. The formed myotubes start producing proteins to form functional myocytes which can be then assembled with known food processing methods (mixing, molding) to form cultured meat (Illustrations have been taken from Servier Medical Art licensed under a Creative Commons Attribution 3.0 Unported License).

The production of cultured meat from SCs is a simple concept which can be briefly described in four steps: (1) satellite cell isolation (2) expansion, (3) differentiation, and (4) assembly of muscle fibers (Figure 1).

Methods and protocols for the identification and isolation of SCs, have already been widely described and only a few milligrams of muscle are now required to isolate a sufficient amount of cells to start a culture (15, 16).

Once SCs are isolated, they need to be expanded *in-vitro* to achieve large cell numbers. SCs are adherent cells, meaning that they need a surface, mimicking an extracellular matrix, for attachment. Flat plastic surfaces coated with a hydrogel are commonly used in satellite cell culture (15, 17). When the required amount of cells is achieved, the differentiation process is initiated. During this step, cells fuse to form myotubes and start expressing proteins characteristic to functional myocytes.

Cell culture with current conventional planar culture systems, presents significant limitations related to their low surface to volume ratio, the lack of pH, gas and metabolite concentration control and is therefore not scalable (18, 19). As a consequence, it is only possible to produce up to 10^11^ cells with these methods (20). Large-scale production requires generation of a significantly higher amount of cells (10^12^-10^13^ cells corresponding to 10–100 kg of meat) while using limited space, time, amount of resources and requiring minimal handling (21). This review aims to discuss the possibility of upscaling cultured meat production with the use of microcarriers, taking into consideration the specific requirements of satellite cells and the specific requirements deriving from the fact that the product needs to be suitable for consumption. The feasibility of a one-step bioprocess will also be discussed.

## Scalability of SC Culture Through the Use of MCs

To address the issue of scalability, three techniques are commonly used for the culture of adherent mammalian cells: (1) culture in aggregates, (2) culture in fixed bed reactors, and (3) culture on microcarriers (MCs). Culture in aggregates consists in the formation of clumps of cells that grow in 3D and serve as anchors for their neighbors (21), whereas MCs are beads composed of various materials, porosities and topographies which provide a surface for anchorage-dependent cells to adhere to McKee and Chaudhry (22). Although very high achieved cell densities have been reported with aggregates (23–25) and in theory, a 3D environment closer to the native environment of the cells is provided, this technique offers little control of aggregates size, resulting in nutrients' and O_2_ gradients inside the aggregates and necrotic cores (22, 26). There are a few reports referring to the aggregate culture of myogenic cells (27–29). However, these were performed with the purpose of sustaining their *in vitro* culture rather than for cell proliferation (doubling times of > 150 h) and were undertaken in static conditions. In addition, Aguanno et al. (30) showed that C2C12 cells cultured in suspension form aggregates that produce extracellular matrix and express markers of quiescent satellite cells, which does not meet the requirement for proliferation (30).

MCs, offering a large surface/volume ratio, are the most promising candidates for upscaling. The suspended microcarriers in the medium offer a 3D culture environment, but the cells still grow on a 2D surface, albeit that the strong curvature of bead surface does affect cell attachment and growth (31–33). Still, the translation from the traditional monolayer culture to a suspension culture is smoother, since the micro-environment of the cells essentially remains the same. They also allow for flexibility in terms of the type of vessel that can be used for scaling-up. Depending on their buoyancy and density, they can be used in stirred-tank, fluidized bed, packed bed and aerated reactors which are commonly used for scaling-up chemical processes and have also been successfully applied to bioprocesses. Microcarrier based bioprocesses also have the advantage of being easier to control and monitor, when compared to fixed bed bioreactor cultures (e.g., hollow fiber or multi-plate), resulting in quality and consistency of the products, as well as cost reduction (34). Lastly, a significant advantage of MC based cultures is that the growth surface provided to the cells can be increased by simply adding new MCs to the culture, as it has been established that cells are able to migrate from bead to bead and populate newly added microcarriers (35–39). This phenomenon, commonly referred to as “bead to bead transfer,” can be explained by two main mechanisms: cells detaching from a confluent MC and reattaching onto other MCs, or cells forming bridges between MCs upon collision (40). It has been shown that successively transferring a small proportion of near-confluent MCs (10–25%) into a new vessel loaded with fresh MCs leads to a decrease of lag phase and an increase of the overall yield (35, 38). Even if using MCs can lead to the formation of cell-loaded MCs aggregates that can inhibit proliferation, adding fresh MCs in combination with adapted agitation have been shown to reduce MCs aggregation (41–43). Although different techniques, such as intermittent stirring, have been implemented to enhance cell transfer and limit clumping of MCs, no robust method for SCs has been reported so far. Since satellite cells do not produce that much ECM as MSCs, which are the cells typically to be reported to be cultured on microcarriers, aggregation of microcarriers is not expected to be a major issue when culturing satellite cells on microcarriers. In our hands, aggregation of microcarriers was less of a problem in the case of satellite cells than has been reported for MSCs (unpublished observations) and indeed, Verbruggen et al., only report aggregation occurring in a microcarrier based culture of satellite cells, when the cell density reaches confluence; when new surface area is introduced to the culture by new microcarrier addition, aggregation abates (39).

Since the first introduction of the MC concept for the culture of adherent cells in 1967 (44), many MCs have been developed and commercialized. Many of them have been discontinued for various reasons, an up to date list with the currently commercially available ones is presented on Supplementary Table 1. MCs have been mostly used for the expansion of cells producing molecules of interest (e.g., monoclonal antibodies, vaccines, proteins) (45) but usually not with the purpose of using the cells as the final product. However, with the latest progress in the field of cell and gene therapy, many efforts (46–51) have been invested in developing MCs for the culture of human stem cells for cell therapies. However, none have been specifically developed for myoblast expansion or meat production.

Microcarriers to be used for meat production should comply with food regulations while also offering an optimal topography and surface chemistry for the target cell type, in this case bovine myoblasts. Ideally, they should also be animal-free to serve the purpose of eliminating the use of animal products throughout the production of cultured meat.

MCs could also serve as nutrient carriers. Essential and/or unstable growth factors, amino acids and nutrients could be loaded and controllably-released from the MC's core, to meet SCs' nutrients demand. This would help reduce the number of medium exchange steps and thus the risk of cell loss or contamination. Perez et al., have succeeded in loading sol-gel derived bioactive glass MCs with basic fibroblast growth factor (FGF-2) and cytochrome c protein, which were sustainably released over a period of several weeks. Mesenchymal stem cells adhered and proliferated to significantly higher levels on the FGF-2 loaded microcarriers when compared to the control (52). Micro-encapsulation and sustained release of bioactive molecules is a field vastly researched for food applications (53, 54) and the same principles can apply for microcarrier based cell culture for meat production. Temperature and pH cues can also be applied to control the *in vitro* release kinetics from loaded microcarriers (55, 56).

## Basic Requirements for SC Adhesion and Proliferation on MCs

**Figure 2:**
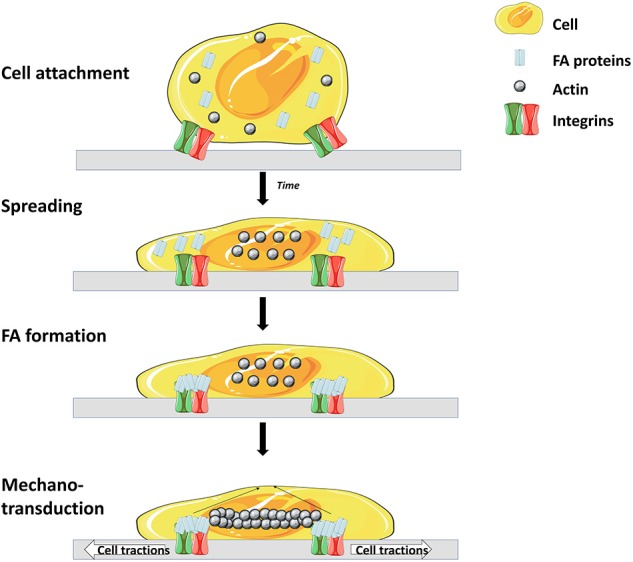
Main steps and molecules involved in cell adhesion to matrix. Cell surface receptors (mostly integrins) interact with specific molecules of the matrix, leading to attachment and spreading of the cell. Attachment is then enhanced through the interaction of focal adhesion (FA) proteins and integrins. Finally, a rearrangement of the cytoskeleton occurs which leads to spreading of the cell over the surface (56) (Illustrations from Goldmann et al. have been recreated with Servier Medical Art, licensed under a Creative Commons Attribution 3.0 Unported License).

Like most mammalian cells SCs are anchorage dependent, hence, cell attachment onto MCs' surfaces is a prerequisite. Cell attachment is a crucial parameter which influences the whole process as a low attachment efficiency will lead to a low expansion yield (57). Cell attachment involves interaction between several cell adhesion molecules (CAMs) and substrates on the surface of the microcarrier (Figure 2) (58).

**Figure 3:**
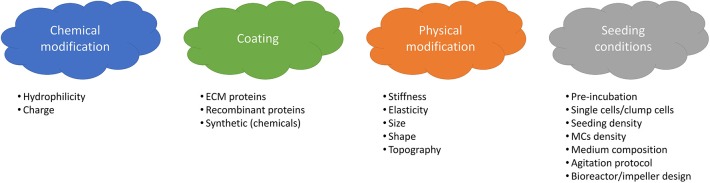
Main variables affecting cell attachment and growth onto MCs.

The integrin family is the main surface receptor family regulating cell adherence (19). They are heterodimeric glycoproteins composed of α and β subunits, each with numerous isoforms (59) and, depending on the subunits expressed, integrins bind to different proteins; for instance α_1_β_1_ has a specific affinity to collagen, α_5_β_1_ to fibronectin and α_v_β_3_ to vitronectin (60). SCs express on their basal surface different integrins, including α_7_β_1_ integrins that bind specifically to laminin (61). In order to enhance cell attachment and proliferation, many efforts have been dedicated to the modification of MCs properties and seeding optimization. Four main strategies are shown in Figure 3.

Coating of the MC surface with extracellular matrix (ECM) proteins, such as collagen, laminin, fibronectin or vitronectin (62) is a widely applied method for the enhancement of cell attachment on MCs. These proteins contain a specific amino acid sequence, so called RGD (for Arginine-Glycine-Aspartate), which is one of the main domains responsible for cell adhesion (19, 63). Using ECM proteins not only has the advantage of enhancing cell attachment, but also provides a more *in-vivo* like environment, resulting in the maintenance of cell functionality and differentiation capacity (19). Wilshut et al. compared attachment, proliferation and differentiation of porcine SCs on several adhesion proteins including Matrigel, gelatin, collagen-1, fibronectin and laminin. Fibronectin and laminin were shown to be more effective in enhancing cell attachment, while laminin and Matrigel provided optimal proliferation and differentiation (64). Dodson et al. performed a similar work with ovine SCs on gelatin, collagen-1, collagen-4, fibronectin, laminin, poly-L-lysine and poly-D-lysine. Best attachment was obtained with fibronectin, whereas optimal proliferation and differentiation were obtained with gelatin (65). Laminin has also been shown to promote cell migration (66) and myoblast proliferation (67). Besides, it has been shown that *in vivo*, the SCs are in contact with the basal membrane of skeletal muscle cells which consists of type IV collagen, laminin, entactin, fibronectin and glycosaminoglycans, such as perlecan (68). Taking into consideration these results and the fact that SCs express laminin (61) and fibronectin receptors (69), the use of laminin or fibronectin as a coating would be promising for enhancing cell attachment as well as proliferation and differentiation. Likewise, the use of other proteins containing the RGD peptide are also promising. Instead of protein coated MCs, conditioning of uncoated microcarriers in a protein containing medium before inoculation can also be effective through adsorption of the protein molecules on the MCs surface (70, 71).

Modification of the MC's surface properties, such as surface charge and hydrophilicity can be achieved by incorporating chemical groups, e.g., amino groups (-NH_2_) or carboxyl groups (-COOH) (19). How surface charge and hydrophilicity influence cell behavior has not been studied in depth, but there is empirical consensus that these factors significantly affect cell attachment and behavior (72). The surface of mammalian cells is known to be negatively charged (73), and therefore, modifications leading to a positively charged surface seem promising. Indeed, Chen et al. (62) observed a lower attachment efficiency of hESCs onto negatively charged compared to positively charged MCs. Similarly, a better attachment on positively compared to negatively charged surfaces was observed by Schneider et al., Lee et al. for a variety of cell types (74–76). Satellite cells have also been shown to successfully attach and grow on positively charged Cytodex 1 microcarriers (39).

Regarding hydrophilicity/hydrophobicity, it is well-established that slightly hydrophilic surfaces lead to better cell attachment (76–79) than hydrophobic (>90°) and superhydrophobic (>150° contact angle) surfaces that have been shown to inhibit mammalian cell adhesion (80). This is mainly due to the fact that hydrophilic surfaces allow for better protein adsorption (81, 82). A super hydrophilic surface of <10° contact angle has also been reported to support CHO cell attachment, however since protein adsorption to superhydrophilic surfaces is very low, the cell attachment in this case can happen only if the cells can directly adhere to the surface chemical groups (83, 84). It becomes clear that, when evaluating the surface chemistry for cell attachment, cell-surface, protein-surface as well as protein-cell interactions should be carefully investigated. For example, Papenburg et al. have observed a better proliferation of C2C12 cells on a more hydrophilic surface, however neither direct correlation between surface wettability and total protein adsorbed nor between total protein adsorbed and cell attachment has been reported. More specifically, cell attachment might be indirectly affected by the wettability through a specific protein ratio adsorbed as well as the conformation of the adsorbed protein. Surfaces presenting both hydrophilic and hydrophobic domains might be preferable for adsorbing different groups of proteins, whereas a mono-phase surface might select a specific kind of protein (85). The degree of adsorption should also be carefully controlled as a weak protein adsorption could result in a lack of binding sites for cell interaction and a strong protein adsorption might affect their conformation. As a general conclusion, it has been shown that surfaces with a moderate hydrophilicity lead to optimal protein adsorption in terms of amount and conformation, and thus optimal attachment and proliferation (84). There is no reason to suspect that satellite cells would behave very differently from other mammalian cells in this context, and therefore, the use of a positively charged with moderately hydrophilic MC surface should favor satellite cell adhesion.

Modification of the physical properties of MCs, such as shape, size, stiffness, elasticity, topography and roughness can also be tuned to enhance satellite cell attachment and proliferation. The definition of an optimal range of these physical properties for this specific cell type is challenging. Stiffness, for instance, is a critical parameter for adherent cell as it can influence cell adhesion (86), protein expression, cytoskeleton modification (87) as well as cell viability (88). Gilbert et al. observed higher engraftment efficiency of SCs when cultured on a poly-ethylene glycol (PEG) gel of muscle-like stiffness (~12 kPa) compared to tissue culture plastic (89). Boonen et al. also observed higher growth rates and sustained proliferation of primary myoblasts on a surface with an elastic modulus of 21 kPa when compared to softer (3 kPa) or stiffer (80 kPa) surfaces (90). It has also been reported that increasing the stiffness from 0.5 to 2 kPa leads to activation/proliferation of mouse myoblasts, whereas at 18 kPa differentiation was induced (91). These findings are in accordance with reported results of 11.5 ± 1.3 kPa stiffness reported for undifferentiated C2C12 cells by Collinsworth et al. (92) and therefore, results from the literature suggest that MCs with a muscle-like stiffness of 2–12 kPa could be beneficial for satellite cell expansion. In order to achieve a desired stiffness, tunable hydrogels have been developed (71) which can offer solutions for controlling satellite cell attachment and proliferation.

Surface topography is another important parameter that affects attachment, proliferation and differentiation of muscle cells. C2C12 cells cultured on a micropatterned surface including pillars showed better cell attachment whereas proliferation and spreading were higher on non-patterned surfaces (85). Better proliferation of C2C12 cells on a randomly oriented nanofibers was observed than on aligned ones, nevertheless, a better fusion and alignment of myoblasts was observed on the latter (93). There is consensus that nanofibrous surfaces, by mimicking the extracellular matrix (ECM), are promoting cell attachment and proliferation (94). In addition to topography, curvature should also be carefully defined since it has been shown to affect the speed (32) as well as the direction and the persistence of hMSCs migration (33). Although other studies have also reported effects of curvature on several cell types including fibroblasts (31, 95), osteoblastic cells (96, 97) and MSCs (31–33), there is a lack of information regarding satellite cells, thus further investigation is still needed. With the increasing development of tools for the fabrication of micro-curved surfaces, more systematic and precise studies should be possible (98).

The size of MCs has also been shown to affect cell behavior. Schmidt et al. reported better cell attachment on larger MCs (1,500 and 3,000 μm) compared to smaller MCs (500 μm). In contrast, a higher growth rate was observed on smaller MCs due to increase in shear stress on the larger ones (99, 100). Nevertheless, the MC diameter should not be <100 μm as most adherent-dependent cells fail to develop their normal morphology and multiply well on sharply curved surfaces (62, 101). It is worth noting here that the development and tailoring of MCs properties for a specific cell type can be very challenging, as traditional material characterization methods used for stiffness, elasticity, topography and roughness measurements are difficult to translate from planar systems to spherical microparticles (19, 100).

Lastly, optimization of the seeding conditions (inoculum and operating parameters) is another way of improving cell attachment (19). The inoculum can be either in the form or single cells or cell clumps, but a few groups have reported that the use of cell clumps leads to heterogeneous distribution of cells onto MCs, resulting in variability in attachment yields (102, 103). There are also discrepancies in the literature (104–107) regarding the optimal cell number per MC to be seeded, that might be traced back to differences in cell types, similar to differences in optimal seeding density on planar systems. On planar culture systems, myoblasts have been cultured at different seeding densities, ranging from 100 to 10,000 cells/cm^2^, with the latter leading to higher growth rate (108). It has to be noted that MCs are often seeded at a slightly higher density than on planar systems, to account for potential losses due to non-attachment (71).

Operational parameters during inoculation also affect cell attachment. The use of dynamic conditions showed positive effect on cell attachment and distribution by increasing cell-MCs contacts (19, 48). However, the seeding density as well as initial MC concentration should be carefully assessed, as cell growth can be negatively affected due to particle collisions (109) and nutrient concentration gradients cause by diffusion limitations (110). Implementing intermittent stirring has also been reported as an efficient strategy and has been widely used for the expansion of stem cells (111–114). Lastly, the bioreactor should also be designed in such a way that shear stress is limited and mixing maximized.

For large scale production, the efficiency of the microcarrier culture, measured as volumetric productivity is an important parameter that needs to be taken into consideration. As microcarriers come in different sizes, shapes and materials, they provide different surface areas per weight and swelling properties. This results in different values of maximum surface area per mL of medium that can be reached with a given MC, defining the maximum volumetric productivity, which is the ultimate efficiency parameter to be carefully considered when up-scaling.

## Scenario Specific Considerations

**Figure 4:**
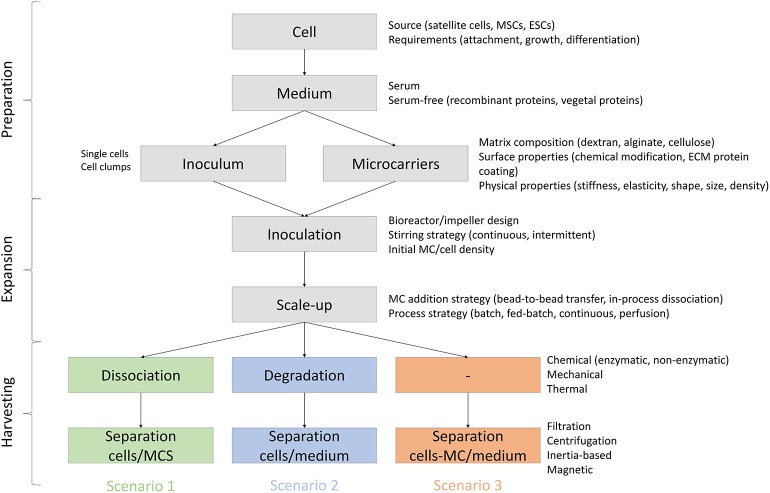
Process requirements and variables for MC based bioprocesses in three scenarios: (1) MCs are serving as a temporary substrate for cell attachment and proliferation and therefore they need to be separated from the cells at some stage of the bioprocess, (2) MCs serve as a temporary substrate for cell proliferation but are degraded or dissolved during the bioprocess, and (3) MCs are embedded in the final product and therefore need to be edible.

Potential attributes of MCs to be used for meat production are reviewed below and are divided based on three different scenarios (Figure 4): (1) MCs are serving only as a temporary substrate for cell attachment and proliferation and therefore they need to be separated from the cells at some stage of the bioprocess, (2) MCs serve as a temporary substrate for cell proliferation but are degraded or dissolved during the bioprocess, and (3) MCs are embedded in the final product and therefore need to be edible.

### Scenario 1: Temporary Microcarriers for SC Proliferation

When microcarriers are used for as temporary substrates for SCs expansion, they need to be removed at the end of the process. There are two important prerequisites in this case: MCs need to (1) provide a high detachment yield (2) and also be easy to separate from the cells.

#### Dissociation

The dissociation of SCs from microcarriers has been shown to be challenging (39, 115). Strategies based on chemical, mechanical and thermal principles have been developed to detach other cell types from MCs while maintaining cell viability, proliferation and differentiation capacity. Chemical detachment consists of enzymatic and non-enzymatic dissociation of cells. The enzymatic detachment is based on proteases, which have the ability to split bonds between amino acids involved in cell attachment, also a very commonly dissociation process used in planar cultures. Proteases are generally used in combination with chelating agents for Ca^2+^ that reduce the ionic strength required for cell binding. The specifics of this protocol are highly dependent on the microcarriers, cells and enzymes used (116, 117). To achieve an animal-free production process, animal-derived proteases can be replaced by recombinant enzymes which have already been proven to efficiently recover cells with high viability while maintaining proliferation and differentiation capacities (118, 119). However, the use of proteases may also lead to proteome (120) and chromatin structure modification (121) which could impact cell stability and subsequent differentiation. For this reason, non-enzymatic techniques have also been researched. Non-enzymatic dissociation agents, such as dextransulphate, N-acetyl-L-cysteine and dithiothreitol, are mimicking enzyme activity that cleaves or degrades MCs coating, if present and have been successfully used for cell detachment from microcarriers.

Mechanical forces are also being used for the detachment of cells from MCs. Katayama et al. showed that pipetting can lead to detachment of epidermal basal cells from Cytodex 3 without the need of trypsin. Rafiq et al., also demonstrated the efficiency of combining the use of trypsin-EDTA with high agitation for the detachment of hMSCs cultured on P102-L MCs (71). Following this, Nienow et al. developed a detailed dissociation protocol based on the Kolmogorov's microscale of turbulence which dictates that to avoid cell damage during dissociation, the size of the biological entity (either the MC size when the cells are attached or the cell size when the cells have detached) has to be smaller than the Kolmogorov scale (λK) (122). With this method, they successfully detached hMSCs from two types of MCs using different bioreactors, medium and enzymes (123). Spier et al. on the other hand, have demonstrated successful detachment and a 90% cell recovery using a vessel with a vibrating plate, which facilitates cell detachment (124). If those techniques would be applicable to SCs is unknown.

The thermal responsivity that certain materials exhibit has also been used to optimize cell detachment from MCs. Thermo-responsive materials have the ability to undergo a discontinuous phase transition and/or morphological modification in response to a variation of temperature (125). By decreasing temperature below the low critical solution temperature (LCST) of the material, the MCs surface becomes very hydrophilic (contact angle <10) leading to cell detachment (126, 127). Many thermo-responsive materials have been used in 2D culture of cells including pluronic (128), an elastin-like polypeptide (129, 130), methylcellulose (50, 126, 131), xyloglucan (132) and hydroxybutyl chitosan (133–136). However, due to its quick phase transition and its LCST at around 32°C, Poly(N-isopropylacrylamide) (PNIPAAm) has retained the most attention so far for temperature induced cell detachment from beads (49, 137–140). Although some researchers report that this detachment method can be time-consuming or less efficient than enzymatic methods (49), better cell viability and ECM protein secretion, as well as better reattachment (141) have been observed.

MCs could also be developed to be fully (core and shell) thermo-responsive. For example, below its LCST (32°C), PNIPAAm is soluble in water and can undergo a gel transition phase above its LCT (126), thus it is theoretically possible to release cells from MCs by collapsing the PNIPAAm gel into a liquid solution.

In a similar way to thermo-responsive polymers, the unique properties of pH, photo or electric current responsive polymers can be harnessed to create smart microcarriers for cell detachment from MCs, however, research on those materials is still at early stages and sometimes difficult to combine with cell culture, thus MCs with such responsivity have not been reported yet (142).

Mechanical and thermal techniques present the advantage that they do not require the use of any dissociation agents which could potentially complicate regulatory requirements. Moreover, chemical techniques require several washing steps before and after dissociation which leads to higher processing times and extensive manipulation of the culture. Usually, a combination of two or even three of the above-mentioned techniques results in lower processing times and tends to limit the side-effects of each of these methods. However, still more in—depth research is required to determine the hydrodynamic conditions in which satellite cells can be detached from MCs without being damaged, as well as to define an optimal combination of techniques for their dissociation.

Lastly, liquid/liquid systems where the cells grow in the interface of the two continuous phases has been demonstrated and present the advantage of facile cell recovery after culture. Several groups (143, 144) showed that mammalian cells can grow in the interface of a fluorocarbon liquid and growth medium. The formation of the two-phase system is based on mutual insolubility and density difference between the phases. Perfluorocarbons have also been successfully shaped in microbeads in stirred-tank bioreactor systems. After the culture period, the cells can be collected from the liquid interface, by inducing coalescence of the emulsion droplets, by removing the proteins that accumulate on their surface, or by centrifugation, thus avoiding the use of proteolytic enzymes. Additionally, fluorocarbon fluids can be oxygenated, allowing for better oxygen transfer in high cell density cultures (145). However, the stirring speeds required to initially prevent the perfluorocarbon particles from coalescing and to resuspend them after sedimentation (needed to replace medium for example) might be prohibitive for some stem cell types.

#### Separation

Once cells have been detached from MCs, they then need to be separated from them. Although many cell/medium separation systems have been developed, only a few are meant to specifically separate cell/MC suspensions. Commercial separation systems are usually based on one of the following four principles: filtration, centrifugation, inertia and magnetism. Dead-end filtration systems have been widely used at small scale, for example nylon filters with mesh sizes of 40–100 μm (111, 146, 147) and have also been developed for relatively larger scale (up to 200 liters): However, as dead-end filtration is generally limited by clogging of filters as the scale increases, more sophisticated systems have prevailed at large scale application. Tangential flow and alternate tangential flow filtration and as well as continuous centrifugal separators are the most used systems, currently. Recently, Moloudi et al. developed an inertia-based device for cells/MCs separation. However, with a filtration rate of 30 ml/min, more efforts are still required to reach an industrially relevant scale (148). Since all of these systems are based on MC size, specific gravity and shape, MCs need to be able to retain their physical properties (integrity, shape, size, density) throughout the culture period.

To overcome issues related to MCs heterogeneity or potential loss of integrity during culture, magnetism can also be used as a separation method. This requires the incorporation of magnetic particles (made from iron, nickel, cobalt or their alloys) into the MCs core. After dissociation of the cells from the surface of the MCs, the introduction of a magnetic field separates the MCs from the cells. This type of microcarriers have not yet been extensively studied and their application has only been reported at a small scale (50 mL culture) (149), however they do seem promising in increasing control over medium exchanges and cell recovery yields, as the challenge of efficiently separating MCs from cells still remains. At present, usually high cell loss percentages are reported by the end of the process, ranging from 15 to 25% (150). On top of that, the risk of foreign material remaining in the retrieved cell pellet and ending up in the food product is high, as commonly, commercial MCs present quite a high variability in size and densities and it is possible that they lose their integrity during the bioprocess, rendering size exclusion methods unsuitable.

The use of a liquid/liquid system or thermally induced collapsing MCs offers significant advantages regarding separation, as it simplifies cell recovery and purification, which can be achieved through repeated washing and centrifugation steps.

When MCs can be completely separated from cells, they will serve as food contact materials, still requiring them to be sufficiently inert so as to not affect consumer health or food quality. Complete separation of non-edible, stable MCs could lead to re-cycle or re-use strategies resulting in reduced waste material and production costs.

### Scenario 2: Non-edible, Degradable Microcarriers

MCs can also serve as a temporary substrate for cell proliferation but instead of being separated at the end of the process they can be degraded at a prior stage. In this case, the dissociation step can be replaced by a microcarrier degradation step to obtain the single cell suspension. Degradation refers to a chemical process that affects chemical composition as well as physical parameters including chain conformation, molecular weight, chain flexibility and cross-linking of a polymer (151). Since the first dextran-based MC, diverse degradable materials have been used for MC production, including polystyrene, cellulose, collagen, gelatin, alginate, chitosan, poly (lactic-co-glycolic acid) (PLGA), polylactide (PLA), or poly(ε-caprolactone) (PCL). Polymers used for their production can be either from natural or synthetic origin, and depending on their properties, they can be degraded in several ways. Degradation can be classified in five categories, based on the factors inducing the process: thermal, chemical, mechanical, photo and biological degradation (151). Bio-chemical and thermal degradation of polymers have been largely investigated in tissue engineering and drug delivery systems, whereas mechanical or photo degradation compatible with cell culture have not yet been reported in the literature.

In the context of cell recovery, degradation of MCs needs to be carefully controlled. The method should be selected in order to be robust, quick (<few hours) and prevent any damage or interaction of the SCs with the degradation products. In addition, MCs' physical properties should remain stable during the expansion phase, as premature degradation of the material will affect proliferation, gene and protein expression of cells as well as the overall control of the bioprocess (152).

SCs have been successfully cultured in many biodegradable hydrogels including alginate (59, 153), fibrin (154, 155), PEG (91, 156), collagen (157) and polyacrylamide (158), however, none of these studies were focused on developing a fast stimulus-degradable material. Usually, a degradation rate matching the tissue skeletal muscle regeneration rate (4–6 weeks) is aimed for (159). Nevertheless, MCs composed of these materials, or of their combinations, could be proven suitable temporary substrates for relatively short duration, large-scale expansion of SCs and stimulus-induced degradation. Accelerated degradation can be achieved with the use of concentrated enzymatic solutions, pH and temperature shifts, with or without concomitant application of mechanical forces (151, 160).

Up to date, only one MC now commercialized by Corning, has been developed with the purpose of being totally and rapidly degraded for cell harvesting (161). It is made of crosslinked polygalacturonic acid (PGA) and can be easily dissolved within 10–20 min using an EDTA solution, which destabilizes the PGA crosslinking in combination with pectinase that digests the polymer. Many other polymers including dextran, cellulose, collagen, pectin or gelatin could be theoretically enzymatically digested in a similar way. For instance, the dextran-based MC Cytodex 1 has not been specifically developed to be degradable, however Lindskog et al. have reported complete dissolution of Cytodex 1 MCs using dextranase while maintaining high cell viability (161). Similarly, the degradation of alginate, which is generally slow *in vivo*, can be accelerated by the use of non-enzymatic chemicals, such as citrate or phosphate. Specifically Voo et al. have shown complete *in vitro* dissolution of 2% and 6% w/v alginate beads in 0.1 M phosphate-buffer solution (pH 7.4) at 37°C after 80 and 240 min, respectively (162). Thermo- and pH responsive degradable beads have also been developed in the context of drug delivery (163, 164). Steinhilber et al. have developed pH-degradable beads, composed of polyglycerol and PEG, which are stable for 2 weeks at 37°C, pH 7.4 and 5% CO_2_ and can be easily degraded in the course of 3 days by lowering the pH to 6.0 while releasing encapsulated NIH3T3 cells with high viability (165).

Of course, the stimuli applied for MC degradation should be compatible with SCs culture requirements, to retain cell function. For instance, Ren et al. reported a dextranase extracted from the marine bacterium *Catenovulum* sp. which presents satisfactory activity (above 80%) at a temperature range of 30–50°C and at a pH ranging from 7.0 to 8.5 (166). Commercially available dextranases, usually fungi derived, are mostly active at acidic pH (5.0–6.0) and higher temperatures (50°C), thus less compatible with cell culture.

Thermal and photo degradation, are also likely to be less suited for cell culture. The high temperatures required to thermally degrade polymers, as well as ultraviolet radiation that is needed to induce photolytic, photo-oxidative and thermo-oxidative reactions (167), resulting in photo-degradation, are also known to cause protein and DNA denaturation and damage (especially UVC: 200–280 nm and UVB: 280–320 nm) (168).

Mechanical forces can be used in combination with chemical degradation (enzymatic or non-enzymatic) to facilitate/accelerate the degradation process and reduce the concentration of enzymes. Increased stirring speeds, shaking or fluidization could serve as such. However, the shear stresses exerted on the cells should be meticulously investigated in order to ensure that cell viability and integrity are maintained.

Overall, results in the literature suggest that there is a variety of materials suitable for degradable MC production, which can be tuned to be stable for the expansion phase and can be *in situ* degraded when a certain stimulus is applied, to allow for the further processing of cells in the differentiation step.

Slowly degrading materials compatible with SC culture could also be used. MCs made of materials that have been developed with the purpose of being bio-chemically degraded *in vivo* in the context of skeletal tissue engineering and drug delivery systems become more relevant in this case (169–172). For instance, Zhou et al. have developed an alginate-fibrin microbead that starts to degrade and release cells 4 days after injection in a calcium phosphate cement scaffold (173).

PLGA and chitosan are also interesting candidates as their degradation rates can be controlled by adjusting the ratio of lactic to glycolic acids and de-acetylation degree, respectively (46, 174). Almeida et al. have developed a curcumin-loaded dual pH and thermo responsive MC. They used pectin, a bio-compatible, biodegradable and non-toxic polysaccharide with pH-responsive properties in combination with PNIPAAm (163). Similarly, Işilkan et al. have developed a pH and thermo-responsive chitosan coated pectin-graft-poly(N,N-diethyl acrylamide) MC (164).

The use of degradable MCs eliminates the need for separation, simplifying the process and resulting in increased cell recovery. Thus, the cell suspension can be washed and used directly for downstream processing. However, it has to be noted that when using degradable MCs, cells are usually released as a sheet/cell-clump, therefore proteolytic enzymes may be additionally needed to promote dissociation into single-cell suspension (161). Depending on the downstream processing of the SCs, however, a single-cell suspension may not be necessarily required and aggregates may be permitted.

### Scenario 3: Edible Microcarriers Embedded in the Final Product

MCs can also be composed of edible materials and be embedded in the final product. As opposed to the previous cases where MCs are considered as a food contact material, in this scenario they should comply with regulations for use as a food ingredient or additive. Indeed, besides supporting cell growth, an edible MC would also be part of the final product and might affect the sensory attributes of the meat product, such as taste, color or texture.

Edible polymers that can be used as substrates for cell expansion are classified into four categories: polysaccharides (e.g., starch, alginate, carrageenan, chitosan, cellulose, carboxymethylcellulose, pectin), polypeptides (e.g., collagen, gelatin, gluten), lipids (e.g., paraffin, shellac), and composites/synthetics (e.g., PGA, PEG) (175). They have been widely used in the food industry as stabilizers, thickeners, coatings and emulsifiers. Cellulose, chitosan and alginate could be good candidates for large-scale expansion of SCs, as they are the most abundant natural polymers and are known for their biocompatibility and biodegradability (176, 177). However, in order to enhance attachment of SCs, incorporation of RGD-containing proteins is required (178), which have not yet received approval for use in food. One patent describing an edible and animal-free MC for engineered meat, proposes the use of pectin coupled with cardosin A, an RGD-containing polypeptide (179). Thus, the use of polypeptides as collagen or gelatin could be more suitable as the tripeptide motif is already naturally present. Using lipid-based MCs could also be an interesting way to bring fatty flavors to the product.

In order to eliminate or limit the effect of the MCs on the sensory profile of the meat, the cells can still be detached and separated from the edible MCs, however, a higher threshold for MCs being present in the recovered cells can be set, allowing for better harvesting yields. Less stringent separation methods, such as separation through sedimentation or centrifugation become more relevant in this context. Edible MCs with controllable degradation properties can also be used and be partially degraded, remaining in the cell harvest for further processing. It should be noted here though, that it is unknown whether remnants of partially degraded MCs could interfere with the differentiation process and would impede the ability of the cells to remodel their environment and fuse into myotubes if seeded in the differentiation scaffold.

The dissociation step can be omitted completely if the MCs are edible. In such case, the edible polymer to be used as cell substrate during the proliferation stage, can also be designed to enhance or introduce desired properties, such as texture, taste or color. For instance, the texture of the final product could be regulated through MCs stiffness. A microcarrier incorporating a hydrogel with specific water retention capacity at high temperatures could be used to enhance juiciness of the cooked product. Additives for a smoked or herb flavor as well as beneficial polyunsaturated fatty acids can also be incorporated through microcarriers. The color of the final product could also be adjusted through the addition of natural food colorings. However, when using MCs that will remain present throughout the process, care should be taken so that their presence doesn't interfere with further processing steps.

Regardless of which polymer is used for MC production, it is essential that its production and processing are well-controlled and comply with food standards regulations. From cross-linking to surface modification of MCs, diverse physical and chemical techniques are used, each one presenting advantages and drawbacks. For instance, physical cross-linking of polymers lead to lower toxicity of the cross-linked material when compared to chemical methods (180). However, toxic compounds are commonly used in many stages of food production and processing, such as the use of pesticides in agriculture, or the use of solvents for oil extraction and the manufacture of food additives. For all edible products, though, including cultured meat, toxicity is based on remaining concentrations in the final product, thus it should be carefully analyzed to meet food grade standards and be safe for human consumption.

Summarizing, edible MCs could be either used as temporary substrate which is either separated or degraded during the process or purposefully used as part of the product that could bring additional sensorial properties. Natural polymers, physically cross-linked seem to be more promising for cultured meat applications as they maintain a better biocompatibility and low toxicity compared to synthetic chemically cross-linked polymers (181).

## Is A One-Step Proliferation/Differentiation Bioprocess Feasible?

In the vast majority of the literature, microcarriers are used for the expansion phase of cell culture, as achieving high cell numbers and specific cell productivity are the ultimate goals for the production of an advanced medicinal product. However, for meat production, the differentiation of SCs into myotubes and subsequently into myofibers is an integral part of the process, which usually happens in a subsequent, separate step. The differentiation phase demands very distinct conditions in terms of nutrients and physical environment. The idea of a simplified bioprocess though, where the same culture system can be used for both phases is very attractive, as it would minimize capital investment in equipment, processing times and cell manipulation. The necessary nutrients can be provided through a switch from a “proliferation medium” to a “differentiation medium,” but providing the physical environment that the cells need in order to differentiate is more challenging. The substrate requirements for the proliferation and differentiation phases are typically different in terms of surface chemistry and topography (88, 91, 93). Stiffness requirements on the other hand shouldn't be difficult to combine for the proliferation and differentiation phase. Although softer substrates are known to retain SC stemness better than stiffer ones which are known to promote differentiation (89), observations on optimal stiffness for proliferation and differentiation often overlap. For example, Engler et al. has shown that culturing mouse myoblasts on a polyacrylamide gel of muscle-like stiffness (~11 kPa) led to better myotube maturity (182), while Boonen et al. have demonstrated better proliferation on a 21 kPa substrate (88). A muscle-like stiffness therefore, in the range of 11–21 kPa could apply for both phases in the presence of other cues.

Torgan et al. have attempted to grow and differentiate SCs on MCs in a stirred-tank bioreactor hypothesizing that simulated microgravity environment would affect the myogenic differentiation. They reported that SCs cultured onto MCs in a microgravity bioreactor expressed less myogenin transcription factor as well as myosin and tropomyosin compared to SCs cultured in a “normal” gravity bioreactor suggesting that mechanical forces affect SCs differentiation (183).

Mechanical stimuli can be transduced by cells via transmembrane proteins into biochemical signals (184) and are also essential to promote protein synthesis and organization into contractile units (8). To promote mechanical stimuli, cells are usually cultured in a gel between anchor points which simulate tendons, thus creating a passive tension which leads to protein production when the tissue compacts (185, 186). This suggests a specifically designed morphology of MCs that allows a similar tension development during tissue formation. In combination to passive forces, different techniques to enhance protein synthesis including application of cyclic stretch (187–189) and electrical stimulation (190, 191) have been attempted. Although Boonen et al. and Kook et al. have not reported any positive effect of cyclic stretching, passive tension seems to be a minimum requirement to promote maturation of muscle cells. Mechanical stimulation of skeletal muscle cells through fluid generated shear stress has been reported in some cases to promote the differentiation process (192–194). High shear stress (5–10 Pa) has been shown to be detrimental to cells, however the effect of lower shear stress ranges (1–1,400 mPa) have been investigated and found to positively influence mechano-transduction in muscle cells. Naskar et al. reported a higher expression of myogenic marker and longer myotubes formed at 16 mPa and a better alignment of cells at 42 mPa (194). Therefore, shear stresses generated during a microcarrier based dynamic culture could be tailored to meet the stimulus required for differentiation, through the tuning of operational parameters, or the design of MCs that allow for the culture of cells onto regions of controlled shear stress, as has been recently reported by Wu et al. (195). Micro-patterned MCs providing topographies favoring myogenic differentiation, such as aligned patterns (93) could also potentially support a one-step bioprocess.

Thus, to support consecutive proliferation and differentiation in one setup, the material used for MCs production should be either tunable *in situ* to meet physical environment requirements for each phase (coating, stiffness, topography and shear stress) or less specific but adapted to both phases. In any case, the use of a non-edible and non-degradable MCs seems unlikely applicable in this situation because, a dissociation and separation step would be needed, which in the case of myotube/myofibers would be more challenging than for individual cells. Following differentiation, MCs could be degraded (or not depending on if the material used is edible), and the produced myofibers (or myofibers-MCs) can be assembled with classic food processing techniques to obtain a product comparable to traditional minced meat.

Besides combining both proliferation and differentiation requirements in one microcarrier, a one-step bioprocess also demands an easy way to maximize productivity of the bioreactor used while maintaining cell performance.

## Concluding Remarks

Based on the MCs physical and chemical properties, several production scenarios for SCs proliferation and differentiation at large-scale are conceivable. Optimization of cell adhesion and expansion on the MCs however, remain a common prerequisite for all scenarios.

Up to date, no MCs have been developed specifically for SCs expansion. The materials for such a MC, as well as the medium composition should be chemically defined to comply with GMP and HACCP standards. Adsorption or coating with recombinant proteins specifically binding to SCs' integrins, such as laminin and fibronectin, tailored substrate stiffness (2–12 kPa) as well as surface properties of the MCs to imitate the SC niche and activate cell proliferation should be taken into account when designing an MC for the expansion of SCs. Chemical modifications to add positive charge to the MC surface, render it moderately hydrophilic or functionalize it with amino groups, would probably be beneficial for SCs attachment. Robust protocols for SCs culture on MCs need to be developed and optimized since the impact of seeding conditions, such as seeding density, type of inoculum and stirring have not been systematically investigated. From current SC culture practices on monolayer, it seems that a positively charged surface with moderate hydrophilicity, protein and peptide coatings and muscle-like stiffness substrates seem to promote the attachment and proliferation of SCs.

In the case of non-edible and non-degradable MCs, cells need to be subsequently detached and separated from MCs. Enzymatic methods have been the most widely used so far for MC cultures and represent the golden standard in cell detachment. However, considering the potential cell damage occurring with this method and the risks associated with cell loss at large-scale production, physical and thermal techniques, based on smart materials have started to be developed and are likely to outperform the use of enzymatic treatments in the future. Following detachment, cells still need to be separated from MCs, and the challenge of achieving high separation yields without MCs residues in the cell pellet, still remains. Although sophisticated single-use filtration systems are already being used in the biopharma industry, for food applications a more straight-forward approach is required to limit production costs. Magnetism, fluidization, vibration and inertia-based separation are currently at early stages of development, but significant work needs to be done for these to be translated into robust devices reliable for production.

In the second scenario, where a degradable MC is used for cell expansion, the dissociation and separation steps can be replaced by a degradation step. Most degradable materials developed so far were designed for *in vivo* degradation and drug release purposes, thus presenting a very slow degradation rate. For large scale bioprocessing of satellite cells, a quick, stimulus induced degradable MC is more applicable.

Edible MCs can also be used, obviating the need to dissociate/separate and degrade the MCs, thus facilitating the production process. Indeed, this third scenario appears to be the most promising for cultured meat production. The use of an edible microcarrier would at least limit the dissociation/degradation/separation steps and can even be tailored to promote organoleptic qualities if embedded in the final product. In the case where microcarriers are not compatible with the differentiation process, edible microcarriers could also be used as a temporary substrate similarly to scenarios 1 and 2 which would limit the risk of non-edible remaining residues. Abundant, cheap, edible and degradable materials, such as alginates, pectins and celluloses seem to be promising candidates for this purpose.

Apart from serving as a passive substrate for cell expansion, MCs can also be engineered to serve as nutrient carriers to the cells or to encapsulate flavors or other substances to enhance the sensorial and nutritional attributes of the final product. Ideally, proliferation and differentiation should be combined in one-step, by providing necessary topographical, mechanical and other cues for differentiation, preferably in a temporal sequence following proliferation.

## Author Contributions

VB wrote the manuscript. PM wrote, edited the manuscript, and provided intellectual input. MP edited, provided intellectual input, and approval for publication.

### Conflict of Interest

VB is employed by Mosa Meat, B.V., a company that aims to commercialize cultured meat. PM is employed by Mosa Meat B.V. MP is co-founder and shareholder of Mosa Meat, B.V.

## References

[1] FAO (ed.). Livestock's Long Shadow - Environmental Issues and Options. Rome (2006).

[2] United Nations World Population Prospects 2019: Data Booklet. New York, NY: Department of Economic Social Affairs (2019). p. 1–25.

[3] OECD/FAO (2016). MEAT. In: OECD-FAO Agricultural Outlook 2016-2025. Paris: OECD publishing.

[4] Tuomisto HL Teixeira De Mattos MJ. Environmental impacts of cultured meat production. Environ Sci Technol. (2011) 45:6117–23. 10.1021/es200130u 21682287

[5] Mattick CS Landis AE Allenby BR Genovese NJ. Anticipatory life cycle analysis of *in vitro* biomass cultivation for cultured meat production in the United States. Environ Sci Technol. (2015) 49:11941–9. 10.1021/acs.est.5b01614 26383898

[6] Lynch J Pierrehumbert R. Climate impacts of cultured meat and beef cattle. Front Sustain Food Syst. (2019) 3:5. 10.3389/fsufs.2019.00005 31535087 PMC6751088

[7] Smetana S Mathys A Knoch A Heinz V Meat alternatives: life cycle assessment of most known meat substitutes. Int J Life Cycle Assess. (2015) 20:1254–67. 10.1007/s11367-015-0931-6

[8] Post MJ. Cultured meat from stem cells: Challenges and prospects. Meat Sci. (2012) 92:297–301. 10.1016/j.meatsci.2012.04.008 22543115

[9] Mead PS Slutsker L Dietz V McCaig LF Bresee JS Shapiro C Food-related illness and death in the United States. J Environ Health. (2000) 62:9–18. 10.3201/eid0505.990502 PMC2627714 10511517

[10] Food and Agriculture Organization of the United Nations “Livestock in food security World,” in World Livestock 2011, ed McLeod A. Rome (2011). p. 130.

[11] Edelman PD McFarland DC Mironov VA Matheny JG. *In vitro*-cultured meat production. Tissue Eng. (2005) 11:659–62. 10.1089/ten.2005.11.659 15998207

[12] Datar I Betti M Possibilities for an *in vitro* meat production system. Innov Food Sci Emerg Technol. (2010) 11:13–22. 10.1016/j.ifset.2009.10.007

[13] Kadim IT Mahgoub O Baqir S Faye B Purchas R Cultured meat from muscle stem cells: a review of challenges and prospects. J Integr Agric. (2015) 14:222–33. 10.1016/S2095-3119(14)60881-9

[14] Mauro A. Satellite cell of skeletal muscle fibers. J Biophys Biochem Cytol. (1961) 9:493–5. 10.1083/jcb.9.2.493 13768451 PMC2225012

[15] Danoviz ME Yablonka-Reuveni Z. Skeletal muscle satellite cells: background and methods for isolation and analysis in a primary culture system. Methods Mol Biol. (2012) 798:21–52. 10.1007/978-1-61779-343-1_2 22130829 PMC3325159

[16] Ding S Swennen GNM Messmer T Gagliardi M Molin DGM Li C. Maintaining bovine satellite cells stemness through p38 pathway. Sci Rep. (2018) 8:1–12. 10.1038/s41598-018-28746-7 30018348 PMC6050236

[17] Stoker M O'Neill C Berryman S Waxman V. Anchorage and growth regulation in normal and virus-transformed cells. Int J Cancer. (1968) 3:683–93. 10.1002/ijc.2910030517 5749478

[18] Oh SKW Chen AK Mok Y Chen X Lim UM Chin A. Long-term microcarrier suspension cultures of human embryonic stem cells. Stem Cell Res. (2009) 2:219–30. 10.1016/j.scr.2009.02.005 19393590

[19] Derakhti S Safiabadi-Tali SH Amoabediny G Sheikhpour M. Attachment and detachment strategies in microcarrier-based cell culture technology: A comprehensive review. Mater Sci Eng C. (2019) 103. 10.1016/j.msec.2019.109782 31349523

[20] Rowley J Abraham E Campbell A Brandwein H Oh S Meeting lot-size challenges of manufacturing adherent cells for therapy. Bioprocess Int. (2012) 10:16–22.

[21] Moritz MSM Verbruggen SEL Post MJ Alternatives for large-scale production of cultured beef: A review. J Integr Agric. (2015) 14:208–16. 10.1016/S2095-3119(14)60889-3

[22] McKee C Chaudhry GR. Advances and challenges in stem cell culture. Colloids Surfaces B Biointerfaces. (2017) 159:62–77. 10.1016/j.colsurfb.2017.07.051 28780462

[23] Abbasalizadeh S Larijani MR Samadian A Baharvand H. Bioprocess development for mass production of size-controlled human pluripotent stem cell aggregates in stirred suspension bioreactor. Tissue Eng Part C Methods. (2012) 18:831–51. 10.1089/ten.tec.2012.0161 22559864

[24] Abecasis B Aguiar T Arnault É Costa R Gomes-Alves P Aspegren A. Expansion of 3D human induced pluripotent stem cell aggregates in bioreactors: bioprocess intensification and scaling-up approaches. J Biotechnol. (2017) 246:81–93. 10.1016/j.jbiotec.2017.01.004 28131858

[25] Davis BM Loghin ER Conway KR Zhang X. Automated closed-system expansion of pluripotent stem cell aggregates in a rocking-motion bioreactor. SLAS Technol. (2018) 23:364–73. 10.1177/2472630318760745 29481762

[26] Egger D Tripisciano C Weber V Dominici M Kasper C. Dynamic cultivation of mesenchymal stem cell aggregates. Bioengineering. (2018) 5:1–15. 10.3390/bioengineering5020048 29921755 PMC6026937

[27] Westerman KA Penvose A Yang Z Allen PD Vacanti CA. Adult muscle “stem” cells can be sustained in culture as free-floating myospheres. Exp Cell Res. (2010) 316:1966–76. 10.1016/j.yexcr.2010.03.022 20381487 PMC2878880

[28] Wei Y Li Y Chen C Stoelzel K Kaufmann AM Albers AE. Human skeletal muscle-derived stem cells retain stem cell properties after expansion in myosphere culture. Exp Cell Res. (2011) 317:1016–27. 10.1016/j.yexcr.2011.01.019 21277299

[29] Hosoyama TG Meyer M Krakora D Suzuki M. Isolation and *in vitro* propagation of human skeletal muscle progenitor cells from fetal muscle. Cell Biol. Int. (2013) 37:191–6. 10.1002/cbin.10026 23319422 PMC3620662

[30] Aguanno S Petrelli C Di Siena S De Angelis L Pellegrini M Naro F. A three-dimensional culture model of reversibly quiescent myogenic cells. Stem Cells Int. (2019) 2019:1–12. 10.1155/2019/7548160 31827532 PMC6885280

[31] Lee SJ Yang S. Micro glass ball embedded gels to study cell mechanobiological responses to substrate curvatures. Rev Sci Instrum. (2012) 83:094302. 10.1063/1.4751869 23020396

[32] Werner M Blanquer SBG Haimi SP Korus G Dunlop JWC Duda GN. Surface curvature differentially regulates stem cell migration and differentiation via altered attachment morphology and nuclear deformation. Adv Sci. (2017) 4:1–11. 10.1002/advs.201600347 28251054 PMC5323878

[33] Werner M Petersen A Kurniawan NA Bouten CVC Cell-perceived substrate curvature dynamically coordinates the direction, speed, and persistence of stromal cell migration. Adv Biosyst. (2019) 3:1900080 10.1002/adbi.201900080 32648723

[34] Rafiq QA Coopman K Hewitt CJ Scale-up of human mesenchymal stem cell culture: Current technologies and future challenges. Curr Opin Chem Eng. (2013) 2:8–16. 10.1016/j.coche.2013.01.005

[35] Ohlson S Branscomb J Nilsson K. Bead-to-bead transfer of chinese hamster ovary cells using macroporous microcarriers. Cytotechnology. (1994) 14:67–80. 10.1007/BF00772197 7765114

[36] Kong D Gentz R Zhang J. Long-term stable production of monocyte-colony inhibition factor (M-CIF) from CHO microcarrier perfusion cultures. Cytotechnology. (1998) 26:131–8. 10.1023/A:1007997412002 22358551 PMC3466677

[37] Hervy M Weber JL Pecheul M Dolley-Sonneville P Henry D Zhou Y. Long term expansion of bone marrow-derived hMSCs on novel synthetic microcarriers in xeno-free, defined conditions. PLoS ONE. (2014) 9:e92120. 10.1371/journal.pone.0092120 24638103 PMC3956887

[38] Rafiq QA Ruck S Hanga MP Heathman TRJ Coopman K Nienow AW Qualitative and quantitative demonstration of bead-to-bead transfer with bone marrow-derived human mesenchymal stem cells on microcarriers: utilising the phenomenon to improve culture performance. Biochem Eng J. (2018) 135:11–21. 10.1016/j.bej.2017.11.005

[39] Verbruggen S Luining D van Essen A Post MJ. Bovine myoblast cell production in a microcarriers-based system. Cytotechnology. (2018) 70:503–12. 10.1007/s10616-017-0101-8 28470539 PMC5851947

[40] Leber J Barekzai J Blumenstock M Pospisil B Salzig D Czermak P Microcarrier choice and bead-to-bead transfer for human mesenchymal stem cells in serum-containing and chemically defined media. Process Biochem. (2017) 59:255–65. 10.1016/j.procbio.2017.03.017

[41] Ferrari C Balandras F Guedon E Olmos E Chevalot I Marc A. Limiting cell aggregation during mesenchymal stem cell expansion on microcarriers. Biotechnol Prog. (2012) 28:780–7. 10.1002/btpr.1527 22374883

[42] Jossen V Schirmer C Mostafa Sindi D Eibl R Kraume M Pörtner R. Theoretical and practical issues that are relevant when scaling up hMSC microcarrier production processes. Stem Cells Int. (2016) 2016. 10.1155/2016/4760414 26981131 PMC4766353

[43] Takahashi I Sato K Mera H Wakitani S Takagi M. Effects of agitation rate on aggregation during beads-to-beads subcultivation of microcarrier culture of human mesenchymal stem cells. Cytotechnology. (2017) 69:503–9. 10.1007/s10616-016-9999-5 27352111 PMC5461241

[44] Van Wezel AL. Growth of cell-strains and primary cells on micro-carriers in homogeneous culture. Nature. (1967) 216:64–5. 10.1038/216064a0 4292963

[45] Phillips BW Horne R Lay TS Rust WL Teck TT Crook JM. Attachment and growth of human embryonic stem cells on microcarriers. J Biotechnol. (2008) 138:24–32. 10.1016/j.jbiotec.2008.07.1997 18771697

[46] Cui Y Liu Y Cui Y Jing X Zhang P Chen X. The nanocomposite scaffold of poly(lactide-co-glycolide) and hydroxyapatite surface-grafted with l-lactic acid oligomer for bone repair. Acta Biomater. (2009) 5:2680–92. 10.1016/j.actbio.2009.03.024 19376759

[47] Shi X Sun L Jiang J Zhang X Ding W Gan Z. Biodegradable polymeric microcarriers with controllable porous structure for tissue engineering. Macromol Biosci. (2009) 9:1211–8. 10.1002/mabi.200900224 19821453

[48] Chen AKL Reuveny S Oh SKW. Application of human mesenchymal and pluripotent stem cell microcarrier cultures in cellular therapy: achievements and future direction. Biotechnol Adv. (2013) 31:1032–46. 10.1016/j.biotechadv.2013.03.006 23531528

[49] Gümüşderelioglu M Çakmak S Timuçin HÖ Çakmak AS. Thermosensitive PHEMA microcarriers: ATRP synthesis, characterization, and usabilities in cell cultures. J Biomater Sci Polym Ed. (2013) 24:2110–25. 10.1080/09205063.2013.827104 23930942

[50] Altomare L Cochis A Carletta A Rimondini L Farè S. Thermo-responsive methylcellulose hydrogels as temporary substrate for cell sheet biofabrication. J Mater Sci Mater Med. (2016) 27:95. 10.1007/s10856-016-5703-8 26984360

[51] Li C Qian Y Zhao S Yin Y Li J. Alginate/PEG based microcarriers with cleavable crosslinkage for expansion and non-invasive harvest of human umbilical cord blood mesenchymal stem cells. Mater Sci Eng C. (2016) 64:43–53. 10.1016/j.msec.2016.03.089 27127027

[52] Perez RA El-Fiqi A Park JH Kim TH Kim JH Kim HW. Therapeutic bioactive microcarriers: co-delivery of growth factors and stem cells for bone tissue engineering. Acta Biomater. (2014) 10:520–30. 10.1016/j.actbio.2013.09.042 24121192

[53] O'Neill GJ Egan T Jacquier JC O'Sullivan M Dolores O'Riordan E. Whey microbeads as a matrix for the encapsulation and immobilisation of riboflavin and peptides. Food Chem. (2014) 160:46–52. 10.1016/j.foodchem.2014.03.002 24799207

[54] Shishir MRI Xie L Sun C Zheng X Chen W Advances in micro and nano-encapsulation of bioactive compounds using biopolymer and lipid-based transporters. Trends Food Sci Technol. (2018) 78:34–60. 10.1016/j.tifs.2018.05.018

[55] Zhou XX Jin L Qi RQ Ma T. Ph-responsive polymeric micelles self-assembled from amphiphilic copolymer modified with lipid used as doxorubicin delivery carriers. R Soc Open Sci. (2018) 5:171654. 10.1098/rsos.171654 29657772 PMC5882696

[56] Matsumoto K Kimura SI Itai S Kondo H Iwao Y. *In vivo* temperature-sensitive drug release system trigged by cooling using low-melting-point microcrystalline wax. J Control Release. (2019) 303:281–8. 10.1016/j.jconrel.2019.04.029 31026549

[57] Bock A Sann H Schulze-Horsel J Genzel Y Reichl U Möhler L. Growth behavior of number distributed adherent MDCK cells for optimization in microcarrier cultures. Biotechnol Prog. (2009) 25:1717–31. 10.1002/btpr.262 19691122

[58] Goldmann WH. Mechanotransduction and focal adhesions. Cell Biol Int. (2012) 36:649–52. 10.1042/CBI20120184 22524451

[59] Rowley JA Madlambayan G Mooney DJ. Alginate hydrogels as synthetic extracellular matrix materials. Biomaterials. (1999) 20:45–53. 10.1016/S0142-9612(98)00107-0 9916770

[60] Barczyk M Carracedo S Gullberg D. Integrins. Cell Tissue Res. (2010) 339:269–80. 10.1007/s00441-009-0834-6 19693543 PMC2784866

[61] Rozo M Li L Fan CM. Targeting β1-integrin signaling enhances regeneration in aged and dystrophic muscle in mice. Nat Med. (2016) 22:889–96. 10.1038/nm.4116 27376575 PMC4974124

[62] Chen AKL Chen X Choo ABH Reuveny S Oh SKW. Critical microcarrier properties affecting the expansion of undifferentiated human embryonic stem cells. Stem Cell Res. (2011) 7:97–111. 10.1016/j.scr.2011.04.007 21763618

[63] Ruoslahti E Pierschbacher MD Argining-glycine-aspartic acid : a versatile *cell* recognition signal minireview. Cell. (1986) 44:517–8. 10.1016/0092-8674(86)90259-X 2418980

[64] Wilschut KJ Haagsman HP Roelen BAJ. Extracellular matrix components direct porcine muscle stem cell behavior. Exp Cell Res. (2010) 316:341–52. 10.1016/j.yexcr.2009.10.014 19853598

[65] Dodson MV Mathison BA Mathison BD. Effects of medium and substratum on ovine satellite cell attachment, proliferation and differentiation *in vitro*. Cell Differ Dev. (1990) 29:59–66. 10.1016/0922-3371(90)90024-Q 2302584

[66] Echtermeyer F Schöber S Pöschl E Von Der Mark H Von Der Mark K Specific induction of cell motility on laminin by α7 integrin. J Biol Chem. (1996) 271:2071–5. 10.1074/jbc.271.4.2071 8567661

[67] Foster RF Thompson JM Kaufman SJ. A laminin substrate promotes myogenesis in rat skeletal muscle cultures: analysis of replication and development using antidesmin and anti-BrdUrd monoclonal antibodies. Dev Biol. (1987) 122:11–20. 10.1016/0012-1606(87)90327-7 3297850

[68] Sanes JR. The basement membrane/basal lamina of skeletal muscle. J Biol Chem. (2003) 278:12601–4. 10.1074/jbc.R200027200 12556454

[69] Bentzinger C Wang YX von Maltzahn J Soleimani VD Yin H Rudnicki MA. Fibronectin regulates Wnt7a signaling and satellite cell expansion. Cell Stem Cell. (2013) 12:75–87. 10.1016/j.stem.2012.09.015 23290138 PMC3539137

[70] Vladkova TG Surface engineered polymeric biomaterials with improved biocontact properties. Int J Polym Sci. (2010) 2010:296094 10.1155/2010/296094

[71] Rafiq QA Coopman K Nienow AW Hewitt CJ. Systematic microcarrier screening and agitated culture conditions improves human mesenchymal stem cell yield in bioreactors. Biotechnol J. (2016) 11:473–86. 10.1002/biot.201400862 26632496 PMC4991290

[72] Meng J Yang G Liu L Song Y Jiang L Wang S Cell adhesive spectra along surface wettability gradient from superhydrophilicity to superhydrophobicity. Sci China Chem. (2017) 60:614–20. 10.1007/s11426-016-9031-8

[73] Weiss L Zeigel R. Cell surface negativity and the binding of positively charged particles. J Cell Physiol. (1971) 77:179–85. 10.1002/jcp.1040770208 4102021

[74] Lee JH Jung HW Kang IK Lee HB. Cell behaviour on polymer surfaces with different functional groups. Biomaterials. (1994) 15:705–11. 10.1016/0142-9612(94)90169-4 7948593

[75] Schneider GB English A Abraham M Zaharias R Stanford C Keller J. The effect of hydrogel charge density on cell attachment. Biomaterials. (2004) 25:3023–8. 10.1016/j.biomaterials.2003.09.084 14967535

[76] Guo S Zhu X Li M Shi L Ong JLT Janczewski D. Parallel control over surface charge and wettability using polyelectrolyte architecture: effect on protein adsorption and cell adhesion. ACS Appl Mater Interfaces. (2016) 8:30552–63. 10.1021/acsami.6b09481 27762557

[77] Dekker A Reitsma K Beugeling T Bantjes A Feijen J van Aken WG. Adhesion of endothelial cells and adsorption of serum proteins on gas plasma-treated polytetrafluoroethylene. Biomaterials. (1991) 12:130–8. 10.1016/0142-9612(91)90191-C 1878448

[78] Goddard JM Hotchkiss JH Polymer surface modification for the attachment of bioactive compounds. Prog Polym Sci. (2007) 32:698–725. 10.1016/j.progpolymsci.2007.04.002

[79] Xu L-C Siedlecki CA Effects of surface wettability and contact time on protein adhesion to biomaterial surfaces. Biomaterials. (2007) 22:3273–83. 10.1016/j.biomaterials.2007.03.032 PMC3671914 17466368

[80] Morán MC Ruano G Cirisano F Ferrari M. Mammalian cell viability on hydrophobic and superhydrophobic fabrics. Mater Sci Eng C. (2019) 99:241–7. 10.1016/j.msec.2019.01.088 30889696

[81] Weathersby PK Horbett TA Hoffman AS. A new method for analysis of the adsorbed plasma protein layer on biomaterial surfaces. Trans Am Soc Artif Intern Organs. (1976) 22:242–51. 951840

[82] Wilson CJ Clegg RE Leavesley DI Pearcy MJ. Mediation of biomaterial-cell interactions by adsorbed proteins: a review. Tissue Eng. (2005) 11:1–18. 10.1089/ten.2005.11.1 15738657

[83] Piret G Galopin E Coffinier Y Boukherroub R Legrand D Slomianny C Culture of mammalian cells on patterned superhydrophilic/superhydrophobic silicon nanowire arrays. Soft Matter. (2011) 7:8642–9. 10.1039/c1sm05838j

[84] Oliveira SM Alves NM Mano JF Cell interactions with superhydrophilic and superhydrophobic surfaces. J Adhes Sci Technol. (2014) 28:843–63. 10.1080/01694243.2012.697776

[85] Papenburg BJ Rodrigues ED Wessling M Stamatialis D Insights into the role of material surface topography and wettability on cell-material interactions. Soft Matter. (2010) 6:4377–88. 10.1039/b927207k

[86] Choquet D Felsenfeld DP Sheetz MP. Extracellular matrix rigidity causes strengthening of integrin- cytoskeleton linkages. Cell. (1997) 88:39–48. 10.1016/S0092-8674(00)81856-5 9019403

[87] Cukierman E Pankov R Stevens DR Yamada KM. Taking cell-matrix adhesions to the third dimension. Science. (2001) 294:1708–12. 10.1126/science.1064829 11721053

[88] Boonen KJM Rosaria-Chak KY Baaijens FPT Van Der Schaft DWJ Post MJ. Essential environmental cues from the satellite cell niche: optimizing proliferation and differentiation. Am J Physiol Cell Physiol. (2009) 296:1338–45. 10.1152/ajpcell.00015.2009 19321742

[89] Gilbert P Havenstrite K Magnusson K Sacco A Leonardi N Kraft P. Substrate elasticity regulates skeletal muscle stem cell self- renewal in culture. Science. (2010) 329:1078–81. 10.1126/science.1191035 20647425 PMC2929271

[90] Boonen Frei H Rossi FM Burt HM Interaction between electrical stimulation, protein coating and matrix elasticity: a complex effect on muscle fibre maturation. Tissue Eng. (2009) 5:601–14. 10.1002/term.289 20603881

[91] Lacraz G Rouleau AJ Couture V Söllrald T Drouin G Veillette N. Increased stiffness in aged skeletal muscle impairs muscle progenitor cell proliferative activity. PLoS ONE. (2015) 10:1–13. 10.1371/journal.pone.0136217 26295702 PMC4546553

[92] Collinsworth AM Zhang S Kraus WE Truskey GA. Apparent elastic modulus and hysteresis of skeletal muscle cells throughout differentiation. Am J Physiol Cell Physiol. (2002) 283:1219–27. 10.1152/ajpcell.00502.2001 12225985

[93] Cha SH Lee HJ Koh WG. Study of myoblast differentiation using multi-dimensional scaffolds consisting of nano and micropatterns. Biomater Res. (2017) 21:1–9. 10.1186/s40824-016-0087-x 28097017 PMC5225639

[94] Mo XM Xu CY Kotaki M Ramakrishna S. Electrospun P(LLA-CL) nanofiber: a biomimetic extracellular matrix for smooth muscle cell and endothelial cell proliferation. Biomaterials. (2004) 25:1883–90. 10.1016/j.biomaterials.2003.08.042 14738852

[95] Park JY Lee DH Lee EJ Lee SH. Study of cellular behaviors on concave and convex microstructures fabricated from elastic PDMS membranes. Lab Chip. (2009) 9:2043–9. 10.1039/b820955c 19568673

[96] Rumpler M Woesz A Dunlop JWC Van Dongen JT Fratzl P. The effect of geometry on three-dimensional tissue growth. J R Soc Interface. (2008) 5:1173–80. 10.1098/rsif.2008.0064 18348957 PMC2495039

[97] Ehrig S Schamberger B Bidan CM West A Jacobi C Lam K. Surface tension determines tissue shape and growth kinetics. Sci Adv. (2019) 5:1–9. 10.1126/sciadv.aav9394 31535019 PMC6739108

[98] Baptista D Teixeira L van Blitterswijk C Giselbrecht S Truckenmüller R. Overlooked? Underestimated? Effects of substrate curvature on cell behavior. Trends Biotechnol. (2019) 37:838–54. 10.1016/j.tibtech.2019.01.006 30885388

[99] Schmidt JJ Jeong J Kong H. The interplay between cell adhesion cues and curvature of cell adherent alginate microgels in multipotent stem cell culture. Tissue Eng Part A. (2011) 17:2687–94. 10.1089/ten.tea.2010.0685 21790303 PMC3204201

[100] Sart S Agathos SN Li Y. Engineering stem cell fate with biochemical and biomechanical properties of microcarriers. Biotechnol Prog. (2013) 29:1354–66. 10.1002/btpr.1825 24124017

[101] Zhou W Seth G Guardia MJ Hu WS Mammalian cell bioreactors. Encycl Ind Biotechnol. (2010) 1–10. 10.1002/9780470054581.eib394

[102] Lock LT Tzanakakis ES. Expansion and differentiation of human embryonic stem cells to endoderm progeny in a microcarrier stirred-suspension culture. Tissue Eng Part A. (2009) 15:2051–63. 10.1089/ten.tea.2008.0455 19196140 PMC2811059

[103] Kehoe DE Jing D Lock LT Tzanakakis ES Ph D. Scalable stirred-suspension bioreactor culture. Tissue Eng Part A. (2010) 16:405–21. 10.1089/ten.tea.2009.0454 19739936 PMC2813185

[104] Butler M Thilly WG MDCK microcarrier cultures: Seeding density effects and amino acid utilization. In Vitro. (1982) 18:213–9. 10.1007/BF02618573 7129475

[105] Ng YC Berry JM Butler M. Optimization of physical parameters for cell attachment and growth on macroporous microcarriers. Biotechnol Bioeng. (1996) 50:627–35. 10.1002/(SICI)1097-0290(19960620)50:6<627::AID-BIT3>3.0.CO;2-M 18627071

[106] Hu WS Meier J Wang DI A mechanistic analysis of the inoculum requirement for the cultivation of mammalian cells on microcarriers. Biotechnology. (2007) 97:52–60. 10.1002/bit.260270507 18553713

[107] Jossen V van den Bos C Eibl R Eibl D. Manufacturing human mesenchymal stem cells at clinical scale: process and regulatory challenges. Appl Microbiol Biotechnol. (2018) 102:3981–94. 10.1007/s00253-018-8912-x 29564526 PMC5895685

[108] Kino-Oka M Chowdhury SR Muneyuki Y Manabe M Saito A Sawa Y. Automating the expansion process of human skeletal muscle myoblasts with suppression of myotube formation. Tissue Eng Part C Methods. (2009) 15:717–28. 10.1089/ten.tec.2008.0429 19284306

[109] Martin C Olmos É Collignon ML De Isla N Blanchard F Chevalot I Revisiting MSC expansion from critical quality attributes to critical culture process parameters. Process Biochem. (2017) 59:231–43. 10.1016/j.procbio.2016.04.017

[110] Panchalingam KM Jung S Rosenberg L Behie LA Bioprocessing strategies for the large-scale production of human mesenchymal stem cells: a review mesenchymal stem/stromal cells - an update. Stem Cell Res Ther. (2015) 6:1–10. 10.1186/s13287-015-0228-5 26597928 PMC4657237

[111] Frauenschuh S Reichmann E Ibold Y Goetz PM Sittinger M Ringe J. A microcarrier-based cultivation system for expansion of primary mesenchymal stem cells. Biotechnol Prog. (2007) 23:187–93. 10.1021/bp060155w 17269687

[112] Serra M Brito C Sousa MFQ Jensen J Tostões R Clemente J. Improving expansion of pluripotent human embryonic stem cells in perfused bioreactors through oxygen control. J Biotechnol. (2010) 148:208–15. 10.1016/j.jbiotec.2010.06.015 20600380

[113] Marinho PAN Vareschini DT Gomes IC Paulsen BDS Furtado DR Castilho LDR. Xeno-free production of human embryonic stem cells in stirred microcarrier systems using a novel animal/human-component-free medium. Tissue Eng Part C Methods. (2013) 19:146–55. 10.1089/ten.tec.2012.0141 22834864

[114] Tozetti PA Caruso SR Mizukami A Fernandes TR da Silva FB Traina F. Expansion strategies for human mesenchymal stromal cells culture under xeno-free conditions. Biotechnol Prog. (2017) 33:1358–67. 10.1002/btpr.2494 28486779

[115] Schnitzler AC Verma A Kehoe DE Jing D Murrell JR Der KA Bioprocessing of human mesenchymal stem/stromal cells for therapeutic use: current technologies and challenges. Biochem Eng J. (2016) 108:3–13. 10.1016/j.bej.2015.08.014

[116] Manousos M Ahmed M Torchio C Wolff J Shibley G Stephens R. Feasibility studies of oncornavirus production in microcarrier cultures. *In vitro*. (1980) 16:507–15. 10.1007/BF02626464 6993346

[117] Carani J Dame M Beals TF Wass JA Growth of three established cell lines on glass microcarriers. Biotechnol Bioeng. (1983) 25:1359–72. 10.1002/bit.260250515 18548765

[118] Rourou S Riahi N Majoul S Trabelsi K Kallel H. Development of an *in situ* detachment protocol of Vero cells grown on Cytodex1 microcarriers under animal component-free conditions in stirred bioreactor. Appl Biochem Biotechnol. (2013) 170:1724–37. 10.1007/s12010-013-0307-y 23737305

[119] Caruso SR Orellana MD Mizukami A Fernandes TR Fontes AM Suazo CAT. Growth and functional harvesting of human mesenchymal stromal cells cultured on a microcarrier-based system. Biotechnol Prog. (2014) 30:889–95. 10.1002/btpr.1886 24574042

[120] Huang HL Hsing HW Lai TC Chen YW Lee TR Chan HT. Trypsin-induced proteome alteration during cell subculture in mammalian cells. J Biomed Sci. (2010) 17:1–10. 10.1186/1423-0127-17-36 20459778 PMC2873939

[121] Kapiszewska M Reddy NMS Lange CS. Trypsin-induced changes in cell shape and chromatin structure result in radiosensitization of monolayer chinese hamster v79 cells. Int J Radiat Biol. (1991) 60:635–46. 10.1080/09553009114552461 1680144

[122] Nienow AW Rafiq QA Coopman K Hewitt CJ A potentially scalable method for the harvesting of hMSCs from microcarriers. Biochem Eng J. (2014) 85:79–88. 10.1016/j.bej.2014.02.005

[123] Nienow AW Hewitt CJ Heathman TRJ Glyn VAM Fonte GN Hanga MP Agitation conditions for the culture and detachment of hMSCs from microcarriers in multiple bioreactor platforms. Biochem Eng J. (2016) 108:24–9. 10.1016/j.bej.2015.08.003

[124] Spier RE Whiteside JP Bolt K. (1977). Trypsinization of BHK 21 monolayer cells grown in two large-scale unit process systems. Biotechnol. Bioeng. 19:1735–8. 10.1002/bit.260191113 922130

[125] Sponchioni M Capasso Palmiero U Moscatelli D. Thermo-responsive polymers: Applications of smart materials in drug delivery and tissue engineering. Mater Sci Eng C. (2019) 102:589–605. 10.1016/j.msec.2019.04.069 31147031

[126] Burdukova E Li H Ishida N O'Shea JP Franks GV. Temperature controlled surface hydrophobicity and interaction forces induced by poly (N-isopropylacrylamide). J Colloid Interface Sci. (2010) 342:586–92. 10.1016/j.jcis.2009.10.049 19913799

[127] Alghunaim A Brink ET Newby BZ. Surface immobilization of thermo-responsive poly(N- isopropylacrylamide) by simple entrapment in a 3- aminopropyltriethoxysilane network. Polymer. (2016) 101:139–50. 10.1016/j.polymer.2016.08.059 28255182 PMC5328667

[128] Higuchi A Aoki N Yamamoto T Miyazaki T Fukushima H Tak TM. Temperature-induced cell detachment on immobilized pluronic surface Akon. J Biomed Mater Res Part A. (2006) 79:380–92. 10.1002/jbm.a.30773 16883586

[129] Mie M Mizushima Y Kobatake E. Novel extracellular matrix for cell sheet recovery using genetically engineered elastin-like protein. J Biomed Mater Res Part B Appl Biomater. (2008) 86:283–90. 10.1002/jbm.b.31019 18161837

[130] Minato A Ise H Goto M Akaike T. Cardiac differentiation of embryonic stem cells by substrate immobilization of insulin-like growth factor binding protein 4 with elastin-like polypeptides. Biomaterials. (2012) 33:515–23. 10.1016/j.biomaterials.2011.09.070 22018385

[131] Chen CH Tsai CC Chen W Mi FL Liang HF Chen SC. Novel living cell sheet harvest system composed of thermoreversible methylcellulose hydrogels. Biomacromolecules. (2006) 7:736–43. 10.1021/bm0506400 16529408

[132] Silva AKA Richard C Ducouret G Bessodes M Scherman D Merten OW. Xyloglucan-derivatized films for the culture of adherent cells and their thermocontrolled detachment: a promising alternative to cells sensitive to protease treatment. Biomacromolecules. (2013) 14:512–9. 10.1021/bm3017737 23244295

[133] Dang JM Sun DDN Shin-Ya Y Sieber AN Kostuik JP Leong KW. Temperature-responsive hydroxybutyl chitosan for the culture of mesenchymal stem cells and intervertebral disk cells. Biomaterials. (2006) 27:406–18. 10.1016/j.biomaterials.2005.07.033 16115680

[134] Chen B Dang J Tan TL Fang N Chen WN Leong KW. Dynamics of smooth muscle cell deadhesion from thermosensitive hydroxybutyl chitosan. Biomaterials. (2007) 28:1503–14. 10.1016/j.biomaterials.2006.11.027 17157377 PMC2376814

[135] Wei YN Wang QQ Gao TT Kong M Yang KK An Y. 3-D culture of human umbilical vein endothelial cells with reversible thermosensitive hydroxybutyl chitosan hydrogel. J Mater Sci Mater Med. (2013) 24:1781–7. 10.1007/s10856-013-4918-1 23526152

[136] Kato A Kan K Ajiro H Akashi M. Development of a rapid *in vitro* tissue deadhesion system using the thermoresponsive sol-gel transition of hydroxybutyl chitosan. J Biomater Sci Polym Ed. (2017) 28:958–73. 10.1080/09205063.2017.1292988 28277005

[137] Park TG Hoffman AS Preparation of large, uniform size temperature-sensitive hydrogel beads. J Polym Sci Part A Polym Chem. (1992) 30:505–7. 10.1002/pola.1992.080300318

[138] Makino K Yamamoto S Fujimoto K Kawaguchi H Ohshima H Surface structure of latex particles covered with temperature-sensitive hydrogel layers. J Colloid Interface Sci. (1994) 166:251–8. 10.1006/jcis.1994.1291

[139] Mee RK Ji HJ Tae GP Swelling induced detachment of chondrocytes using RGD-modified poly(N-isopropylacrylamide) hydrogel beads. Biotechnol Prog. (2002) 18:495–500. 10.1021/bp020287z 12052065

[140] Nguyen LTB Odeleye AOO Chui CY Baudequin T Cui Z Ye H. Development of thermo-responsive polycaprolactone macrocarriers conjugated with Poly(N-isopropyl acrylamide) for cell culture. Sci Rep. (2019) 9:3477. 10.1038/s41598-019-40242-0 30837639 PMC6401373

[141] Tamura A Nishi M Kobayashi J Nagase K Yajima H Yamato M. Simultaneous enhancement of cell proliferation and thermally induced harvest efficiency based on temperature-responsive cationic copolymer-grafted microcarriers. Biomacromolecules. (2012) 13:1765–73. 10.1021/bm300256e 22616950

[142] Tavassoli H Alhosseini SN Tay A Chan PPY Weng Oh SK Warkiani ME. Large-scale production of stem cells utilizing microcarriers: a biomaterials engineering perspective from academic research to commercialized products. Biomaterials. (2018) 181:333–46. 10.1016/j.biomaterials.2018.07.016 30098569

[143] Giaever I Keese CR. Behavior of cells at fluid interfaces. Proc Natl Acad Sci USA. (1983) 80:219–22. 10.1073/pnas.80.1.219 6571995 PMC393343

[144] Hanga MP Murasiewicz H Pacek AW Nienow AW Coopman K Hewitt CJ. Expansion of bone marrow-derived human mesenchymal stem/stromal cells (hMSCs) using a two-phase liquid/liquid system. J Chem Technol Biotechnol. (2017) 92:1577–89. 10.1002/jctb.5279 28706339 PMC5485050

[145] Pilarek M Grabowska I Ciemerych MA Dabkowska K Szewczyk KW. Morphology and growth of mammalian cells in a liquid/liquid culture system supported with oxygenated perfluorodecalin. Biotechnol Lett. (2013) 35:1387–94. 10.1007/s10529-013-1218-2 23666427 PMC3730094

[146] Weber C Kassem M Pohl S Pörtner R Wallrapp C Peter G. Expansion and Harvesting of hMSC-TERT. Open Biomed Eng J. (2007) 1:38–46. 10.2174/1874120700701010038 19662126 PMC2701074

[147] Goh TKP Zhang ZY Chen AKL Reuveny S Choolani M Chan JKY. Microcarrier culture for efficient expansion and osteogenic differentiation of human fetal mesenchymal stem cells. Biores Open Access. (2013) 2:84–97. 10.1089/biores.2013.0001 23593561 PMC3620494

[148] Moloudi R Oh S Yang C Teo KL Lam ATL Warkiani ME. Inertial-Based Filtration Method for Removal of Microcarriers from Mesenchymal Stem Cell Suspensions. Sci Rep. (2018) 8:1–10. 10.1038/s41598-018-31019-y 30127526 PMC6102204

[149] Lin CY Huang CH Wu YK Cheng NC Yu J. Maintenance of human adipose derived stem cell (hASC) differentiation capabilities using a 3D culture. Biotechnol Lett. (2014) 36:1529–37. 10.1007/s10529-014-1500-y 24658740

[150] Billig D Clark JM Ewell AJ Carter CM Gebb C. The separation of harvested cells from microcarriers: a comparison of methods. Dev Biol Stand. (1983) 55:67–75. 6677541

[151] Jasso-Gastinel CF Soltero-Martínez JFA Mendizábal E Introduction: modifiable characteristics and applications. In: Jasso-Gastinel CF Kenny JM editors. Modification of Polymer Properties. William Andrew Applied Science Publisher (2017). p. 1–21.

[152] Sung HJ Meredith C Johnson C Galis ZS. The effect of scaffold degradation rate on three-dimensional cell growth and angiogenesis. Biomaterials. (2004) 25:5735–42. 10.1016/j.biomaterials.2004.01.066 15147819

[153] Wang L Cao L Shansky J Wang Z Mooney D Vandenburgh H. Minimally invasive approach to the repair of injured skeletal muscle with a shape-memory scaffold. Mol Ther. (2014) 22:1441–9. 10.1038/mt.2014.78 24769909 PMC4435589

[154] Page RL Malcuit C Vilner L Vojtic I Shaw S Hedblom E. Restoration of skeletal muscle defects with adult human cells delivered on fibrin microthreads. Tissue Eng Part A. (2011) 17:2629–40. 10.1089/ten.tea.2011.0024 21699414

[155] Chiron S Tomczak C Duperray A Lainé J Bonne G Eder A. Complex interactions between human myoblasts and the surrounding 3D fibrin-based matrix. PLoS ONE. (2012) 7:2–9. 10.1371/journal.pone.0036173 22558372 PMC3338613

[156] Salimath AS García AJ. Biofunctional hydrogels for skeletal muscle constructs. J Tissue Eng Regen Med. (2016) 10:967–76. 10.1002/term.1881 24616405

[157] Sakar MS Neal D Boudou T Borochin MA Li Y Weiss R. Formation and optogenetic control of engineered 3D skeletal muscle bioactuators. Lab Chip. (2012) 12:4976–85. 10.1039/c2lc40338b 22976544 PMC3586563

[158] Serena E Zatti S Reghelin E Pasut A Cimetta E Elvassore N. Soft substrates drive optimal differentiation of human healthy and dystrophic myotubes. Integr Biol. (2010) 2:193–201. 10.1039/b921401a 20473399

[159] Gates C Huard J Management of skeletal muscle injuries in military personnel. Oper Tech Sports Med. (2005) 13:247–56. 10.1053/j.otsm.2006.01.012

[160] Alexis F Factors affecting the degradation and drug-release mechanism of poly(lactic acid) and poly[(lactic acid)-co-(glycolic acid)]. Polym Int. (2005) 54:36–46. 10.1002/pi.1697

[161] Rodrigues AL Rodrigues CAV Gomes AR Vieira SF Badenes SM Diogo MM. Dissolvable microcarriers allow scalable expansion and harvesting of human induced pluripotent stem cells under xeno-free conditions. Biotechnol J. (2019) 14:1–12. 10.1002/biot.201800461 30320457

[162] Voo WP Lee BB Idris A Islam A Tey BT Chan ES Production of ultra-high concentration calcium alginate beads with prolonged dissolution profile. RSC Adv. (2015) 5:36687–95. 10.1039/C5RA03862F

[163] Almeida EAMS Bellettini IC Garcia FP Farinácio MT Nakamura CV Rubira AF. Curcumin-loaded dual pH- and thermo-responsive magnetic microcarriers based on pectin maleate for drug delivery. Carbohydr Polym. (2017) 171:259–66. 10.1016/j.carbpol.2017.05.034 28578962

[164] Işiklan N Tokmak S. Development of thermo/pH-responsive chitosan coated pectin-graft-poly(N,N-diethyl acrylamide) microcarriers. Carbohydr Polym. (2019) 218:112–25. 10.1016/j.carbpol.2019.04.068 31221312

[165] Steinhilber D Rossow T Wedepohl S Paulus F Seiffert S Haag R. A microgel construction kit for bioorthogonal encapsulation and pH-controlled release of living cells. Angew Chem Int Ed. (2013) 52:13538–43. 10.1002/anie.201308005 24288142

[166] Ren W Cai R Yan W Lyu M Fang Y Wang S. Purification and characterization of a biofilm-degradable dextranase from a marine bacterium. Mar Drugs. (2018) 16:1–16. 10.3390/md16020051 29414837 PMC5852479

[167] Yousif E Haddad R. Photodegradation and photostabilization of polymers, especially polystyrene: review. Springerplus. (2013) 2:1–32. 10.1186/2193-1801-2-398 25674392 PMC4320144

[168] Pattison DI Davies MJ. Actions of ultraviolet light on cellular structures. In: Bignold LP editor. Cancer: Cell Structures, Carcinogens and Genomic Instability. Birkhäuser (2006). 131–157. 10.1007/3-7643-7378-4_6 16383017

[169] Park JH Pérez RA Jin GZ Choi SJ Kim HW Wall IB. Microcarriers designed for cell culture and tissue engineering of bone. Tissue Eng Part B Rev. (2013) 19:172–90. 10.1089/ten.teb.2012.0432 23126371

[170] Li B Wang X Wang Y Gou W Yuan X Peng J. Past, present, and future of microcarrier-based tissue engineering. J Orthop Transl. (2015) 3:51–7. 10.1016/j.jot.2015.02.003 30035040 PMC5982391

[171] Fu C Yang X Tan S Song L. Enhancing cell proliferation and osteogenic differentiation of MC3T3-E1 pre-osteoblasts by BMP-2 delivery in graphene oxide-incorporated PLGA/HA biodegradable microcarriers. Sci Rep. (2017) 7:1–13. 10.1038/s41598-017-12935-x 28970533 PMC5624967

[172] Choe G Park J Park H Lee JY. Hydrogel biomaterials for stem cell microencapsulation. Polymers. (2018) 10:1–17. 10.3390/polym10090997 30960922 PMC6403586

[173] Zhou H Xu HHK. The fast release of stem cells from alginate-fibrin microbeads in injectable scaffolds for bone tissue engineering. Biomaterials. (2011) 32:7503–13. 10.1016/j.biomaterials.2011.06.045 21757229 PMC3159408

[174] Freier T Koh HS Kazazian K Shoichet MS. Controlling cell adhesion and degradation of chitosan films by N-acetylation. Biomaterials. (2005) 26:5872–8. 10.1016/j.biomaterials.2005.02.033 15949553

[175] Shit SC Shah PM Edible polymers: challenges and opportunities. J Polym. (2014) 2014:1–13. 10.1155/2014/427259

[176] Chang C Zhang L Cellulose-based hydrogels: present status and application prospects. Carbohydr Polym. (2011) 84:40–53. 10.1016/j.carbpol.2010.12.023

[177] Ahmadi F Oveisi Z Samani M Amoozgar Z. Chitosan based hydrogels: characteristics and pharmaceutical applications. Res Pharm Sci. (2015)10:1–16. 26430453 PMC4578208

[178] Gasperini L Mano JF Reis RL. Natural polymers for the microencapsulation of cells. J R Soc Interface. (2014) 11:20140817. 10.1098/rsif.2014.0817 25232055 PMC4191114

[179] Marga FS Brendan P Forgacs G Forgacs A Edible and Animal-Product-Free Microcarriers for Engineered Meat. PCT Int. Appl. U.S. Patent No WO2015038988A1. (2015) p. 33.

[180] Liu LS Kost J Yan F Spiro RC Hydrogels from biopolymer hybrid for biomedical, food, and functional food applications. Polymers. (2012) 4:997–1011. 10.3390/polym4020997

[181] Ali A Ahmed S. Recent advances in edible polymer based hydrogels as a sustainable alternative to conventional polymers. J Agric Food Chem. (2018) 66:6940–67. 10.1021/acs.jafc.8b01052 29878765

[182] Engler AJ Griffin MA Sen S Bönnemann CG Sweeney HL Discher DE Myotubes differentiate optimally on substrates with tissue-like stiffness: Pathological implications for soft or stiff microenvironments. J Cell Biol. (2004) 166:877–87. 10.1083/jcb.200405004 15364962 PMC2172122

[183] Torgan CE Burge SS Collinsworth AM Truskey GA Kraus WE. Differentiation of mammalian skeletal muscle cells cultured on microcarrier beads in a rotating cell culture system. Med Biol Eng Comput. (2000) 38:583–90. 10.1007/BF02345757 11094818

[184] Tarbell JM Shi Z-D. Effect of the glycocalyx layer on transmission of interstitial flow shear stress to embedded cells. Biomech Model Mechanobiol. (2013) 12:111–21. 10.1007/s10237-012-0385-8 22411016 PMC3394897

[185] Morgan JR Yarmush ML Vandenburgh H Shansky J Del Tatto M Chromiak J Organogenesis of skeletal muscle in tissue culture. Tissue Eng. (2003) 217–26. 10.1385/0-89603-516-6:217 21370179

[186] Vandenburgh HH Karlisch P. Longitudinal growth of skeletal myotubes *in vitro* in a new horizontal mechanical cell stimulator. Vitr Cell Dev Biol. (1989) 25:607–16. 10.1007/BF02623630 2753848

[187] Powell CA Smiley BL Mills J Vandenburgh HH. Mechanical stimulation improves tissue-engineered human skeletal muscle. Am J Physiol Cell Physiol. (2002) 283:1557–65. 10.1152/ajpcell.00595.2001 12372817

[188] Kook SH Son YO Choi KC Lee HJ Chung WT Hwang IH. Cyclic mechanical stress suppresses myogenic differentiation of adult bovine satellite cells through activation of extracellular signal-regulated kinase. Mol Cell Biochem. (2008) 309:133–41. 10.1007/s11010-007-9651-y 18008139

[189] Boonen KJM Langelaan MLP Polak RB van der Schaft DWJ Baaijens FPT Post MJ. Effects of a combined mechanical stimulation protocol: value for skeletal muscle tissue engineering. J Biomech. (2010) 43:1514–21. 10.1016/j.jbiomech.2010.01.039 20189177

[190] Fujita H Nedachi T Kanzaki M. Accelerated de novo sarcomere assembly by electric pulse stimulation in C2C12 myotubes. Exp Cell Res. (2007) 313:1853–65. 10.1016/j.yexcr.2007.03.002 17425954

[191] Langelaan MLP Boonen KJM Rosaria-Chak KY van der Schaft DWJ Post MJ Baaijens FPT. Advanced maturation by electrical stimulation: Differences in response between C2C12 and primary muscle progenitor cells. Tissue Eng. (2011) 5:529–39. 10.1002/term.345 21695794

[192] Juffer P Bakker AD Klein-Nulend J Jaspers RT. Mechanical loading by fluid shear stress of myotube glycocalyx stimulates growth factor expression and nitric oxide production. Cell Biochem Biophys. (2014) 69:411–9. 10.1007/s12013-013-9812-4 24402674

[193] Kurth F Franco-Obregón A Casarosa M Küster SK Wuertz-Kozak K Dittrich PS. Transient receptor potential vanilloid 2-mediated shear-stress responses in C2C12 myoblasts are regulated by serum and extracellular matrix. FASEB J. (2015) 29:4726–37. 10.1096/fj.15-275396 26207028

[194] Naskar S Kumaran V Basu B On the origin of shear stress induced myogenesis using PMMA based lab-on-chip. ACS Biomater Sci Eng. (2017) 3:1154–71. 10.1021/acsbiomaterials.7b00206 33429590

[195] Wu CY Stoecklein D Kommajosula A Lin J Owsley K Ganapathysubramanian B. Shaped 3D microcarriers for adherent cell culture and analysis. Microsystems Nanoeng. (2018) 4:21. 10.1038/s41378-018-0020-7 31057909 PMC6220171

