# Supplementary material for: Microcarriers for Upscaling Cultured Meat Production
Source: Front Nutr. 2020 Feb 20;7:10. doi: 10.3389/fnut.2020.00010 (PMC7045063; doi:10.3389/fnut.2020.00010)
Supplement: Supplementary file 1 [file Table_1.DOCX]

| **Microcarrier** | **Manufacturer** | **Matrix** | **Surface coating** | **Surface charge** | **Diameter (µm)** | **Specific density** | **Porosity** | **Specific surface area (cm2/g DW)** |
| --- | --- | --- | --- | --- | --- | --- | --- | --- |
| ***Animal-free microcarriers*** | | | | | | | | |
| Cytodex 1 | GE Healthcare | Cross-linked dextran | DEAE | + | 147 - 248 | 1.03 | Non-porous | 4,400 |
| Cytopore 1 & 2 | GE Healthcare | Cross-linked cotton cellulose | DEAE | + | 200 - 280 | 1.03 | >90%;  pore size: 30 µm | 11,000 |
| Cytoline 1 | GE Healthcare | Polystyrene | None | - | Lentil shaped; Length: 1.7-2.5 mm Thickness: 0.4 - 1.1mm | 1.32 | Pore size: 10 – 400μm | >3,000 |
| Cytoline 2 | GE healthcare | HDPE*/Silica | None | - | Lentil shaped; Length: 1.7-2.5 mm Thickness: 0.4 - 1.1mm | 1.03 | Pore size: 10 – 400μm | >1,000 |
| Enhanced attachment | Corning | Polystyrene | CellBIND | None | 125 - 212 | 1.02 | Non-porous | 360 |
| Hillex® | SoloHill | Modified polystyrene | Cationic trimethyl ammonium | + | 160 - 200 | 1.09-1.15 | Non-porous | 515 |
| Plastic | SoloHill | Cross-linked polystyrene | None | None | 90 - 150 | 1.02-1.03 | Non-porous | 360 |
| Plastic Plus | SoloHill | Cross-linked polystyrene | None | + | 125 - 212 | 1.034 -1.046 | Non-porous | 360 |
| Star Plus | SoloHill | Cross-linked polystyrene | None | + | 125 - 212 | 1.02-1.03 | Non-porous | 360 |
| Untreated | Corning | Polystyrene | None | None | 125-212 | 1.026 | Non-porous | 360 |
| Dissolvable | Corning | Cross-linked PGA | Synthemax II | None | 200 - 300 | 1.02-1.03 | Non-porous | 5,000 |
| Synthemax II | Corning | Polystyrene | Synthemax II | None | 125 - 212 | 1.02 | Non-porous | 360 |
| ***Animal derived protein-coated microcarriers*** | | | | | | | | |
| Collagen | SoloHill | Polystyrene | Type I porcine collagen | None | 125 - 212 | 1.02 | Non-porous | 480 |
| Cultispher-G® | Percell | Type I porcine gelatin | None | None | 130 - 380 | 1.02 - 1.04 | 50% ;  pore size: 50 µm | 15,000 |
| Cultispher-S® | Percell | Gelatin | None | None | 130 - 380 | 1.02 - 1.04 | 50% ;  pore size: 50 µm | 15,000 |
| Cytodex 3 | GE Healthcare | Cross-linked dextran | Type I porcine collagen | None | 141 - 211 | 1.04 | Non-porous | 2,700 |
| FACT III | SoloHill | Polystyrene | Cationic type I porcine collagen | + | 125 - 212 | 1.02 | Non-porous | 480 |
| Collagen Coated | Corning | Polystyrene | Type I collagen | None | 125 - 212 | 1.026 | Non porous | 360 |
| Dissolvable | Corning | Cross-linked PGA | Denatured Collagen | None | 200 - 300 | 1.02-1.03 | Non porous | 5,000 |
| SphereCol® | Advanced BioMatrix | Polystyrene | Type I human collagen (VitroCol®) | None | 125 - 212 | 1.03 | Non-porous | 360 |
| ***Recombinant protein-coated microcarriers*** | | | | | | | | |
| ProNectin® F | SoloHill | Polystyrene | RGD containing peptide | None | 125 - 212 | 1.02 | Non-porous | 360 |

*High density poly ethylene
